# Targeting ferroptosis in major digestive system malignancies: regulatory networks and therapeutic opportunities

**DOI:** 10.3389/fcell.2026.1945918

**Published:** 2026-10-29

**Authors:** Xiaoguang Qin, Qingna Wen, Yufei Yang, Kai Sun, Wenjing Guan, Lingna Ma, Penghui Sun, Ming Zhang, Yana Wu, Linzhu Li, Kaikai Wang

**Affiliations:** 1 College of Acupuncture-Moxibustion and Tuina, Gansu University of Chinese Medicine, Lanzhou, Gansu Province, China; 2 Higher Vocational and Technical College, Henan Institute of Science and Technology, Xinxiang, Henan Province, China; 3 School of Public Health, Gansu University of Chinese Medicine, Lanzhou, Gansu Province, China; 4 The First Clinical Medical School, Henan University of Chinese Medicine, Zhengzhou, Henan Province, China; 5 Department of Hematology and Oncology, Henan Integrative Medicine Hospital, Zhengzhou, Henan Province, China; 6 College of Pharmacy, Fujian University of Traditional Chinese Medicine, Fuzhou, Fujian, China; 7 Department of Acupuncture and Tuina, Baicheng County Hospital of Traditional Chinese Medicine, Baicheng, China; 8 Department of Oncology, Affiliated Hospital of Gansu University of Chinese Medicine, Lanzhou, Gansu, China; 9 Clinical College of Traditional Chinese Medicine, Gansu University of Chinese Medicine, Lanzhou, Gansu Province, China

**Keywords:** digestive system malignancies, ferroptosis, lipid peroxidation, targeted therapy, tumor microenvironment

## Abstract

Ferroptosis is an iron-dependent form of regulated cell death that is associated with the accumulation of lipid peroxides, particularly in polyunsaturated fatty acids (PUFAs)-containing cellular membranes. Ferroptosis is initiated by aberrant iron metabolism, intense reactive oxygen species (ROS) production, oxidative damage to membrane structures and disruption of the cystine/glutamate antiporter system (System Xc^−^)–glutathione (GSH)–glutathione peroxidase 4 (GPX4) antioxidant pathway. Emerging evidence increasingly recognizes ferroptosis as a crucial mediator of tumor progression and response to therapy in digestive system malignancies. Aberrant iron homeostasis, lipid metabolic reprogramming, redox imbalance and remodeling of the tumor microenvironment (TME) are common events found in digestive system malignancies. This structured narrative review primarily focuses on colorectal cancer (CRC), hepatocellular carcinoma (HCC), gastric cancer (GC), and pancreatic ductal adenocarcinoma (PDAC) because ferroptosis regulatory mechanisms have been best defined for these cancers with respect to tumor-intrinsic regulation, metabolic vulnerability, antioxidant dependency, TME interactions and therapeutic responses. Additional evidence assessing whether the regulatory principles of ferroptosis extend to additional digestive system malignancies such as esophageal squamous-cell carcinoma and extrahepatic cholangiocarcinoma is also presented. Collectively, these modifications contribute to ferroptosis sensitivity as well as tumor growth, treatment resistance and immune evasion. Ferroptosis is not simply regulated by a single linear pathway, but rather orchestrated through multilayered regulatory networks involving transcriptional and epigenetic control, metabolic rewiring, immune modulation, exosome-mediated communication and extensive crosstalk between numerous signaling pathways. The application of ferroptosis inducers, nano-platform delivery systems, TME-responsive methodology and multimodal combination schemes indicates emerging therapeutic approaches with potential translational implications for digestive system malignancies. Nonetheless, clinical translation is limited by mechanistic complexity, tumor heterogeneity, incompletely defined resistance mechanisms, uncertainty of reliable predictive biomarkers and unaddressed biosafety issues with nanoplatforms. Thus, this Review frames current evidence within a three-tier mechanistic-to-translational framework that connects a common iron–lipid–redox vulnerability axis possessing tumor-type-specific resistance programs and biomarker-directed therapeutic prioritization to delineate context-dependent ferroptosis vulnerabilities and priorities for future clinical evaluation.

## Graphical Abstract

**Figure:**
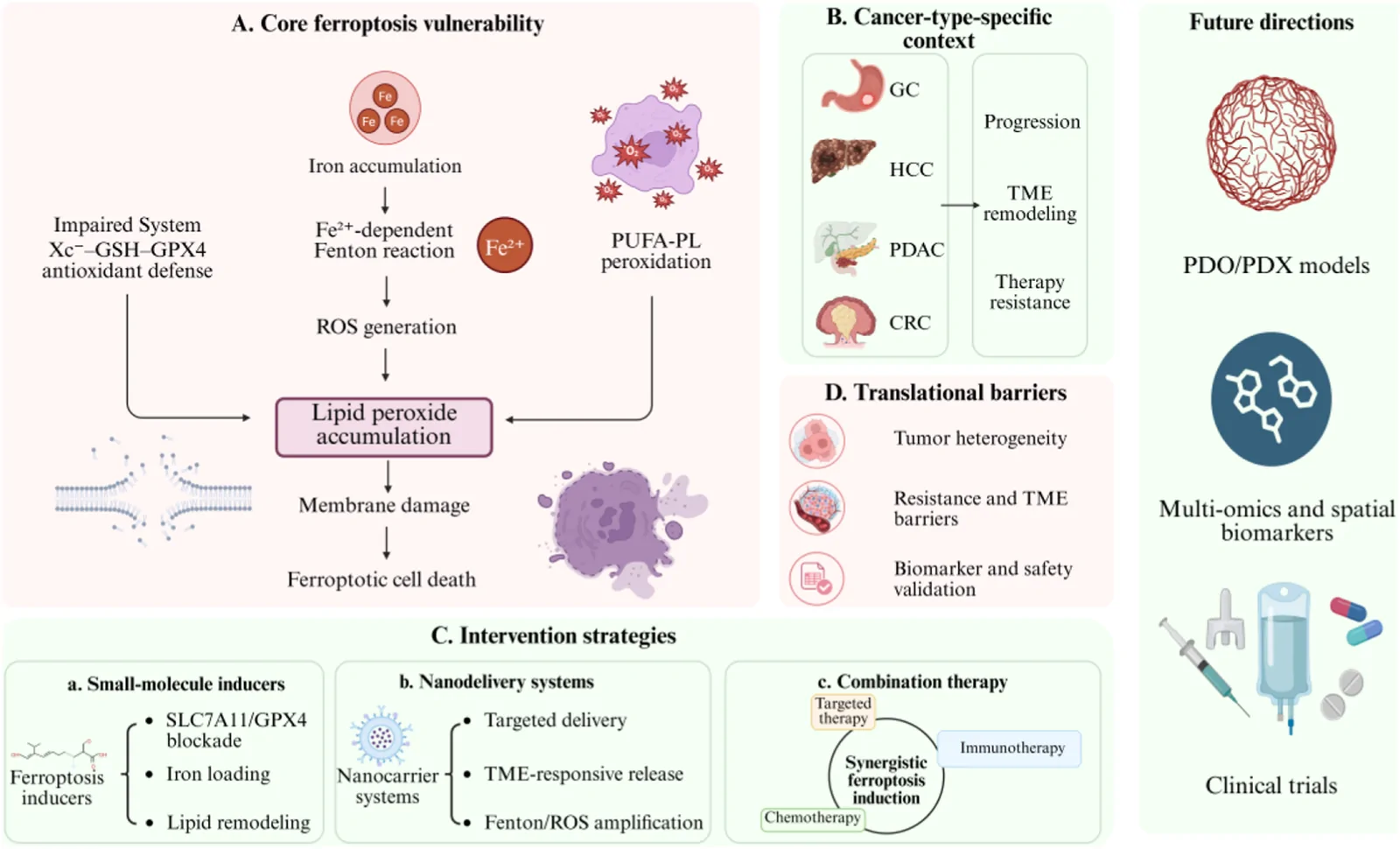

## 1 Introduction

Digestive system malignancies, particularly CRC, GC, HCC, and PDAC, remain a major global health burden. Despite substantial advances in surgical resection, chemotherapy, radiotherapy, molecularly targeted therapy and immunotherapy, durable clinical benefits remain limited for many patients, largely due to marked tumor heterogeneity, frequent recurrence and metastasis, and therapeutic resistance (Quan et al., 2024). Tumor cells can evade therapeutic pressure through multiple mechanisms, including metabolic reprogramming, redox homeostasis adaptation, enhanced antioxidant defenses, and dynamic remodeling of the TME, particularly in advanced or recurrent disease (Liudahl et al., 2021; Shen et al., 2025). These limitations underscore the need to identify alternative regulated cell-death programs and therapeutically actionable vulnerabilities in digestive system cancers.

Ferroptosis is a novel form of regulated cell death that is iron-dependent and mechanistically different from classical apoptosis, necrosis and autophagy. Its generation happens when lipid peroxidation outstrips cellular antioxidant capabilities leading to oxidative destruction of membrane phospholipids and loss of integrity. Elevated iron absorption leads to intracellular Fe^2+^ accumulation, which, in the presence of O_2_, accelerates Fenton reactions and generates massive amounts of ROS. PUFA-containing phospholipids (PUFA-PLs) are the principal substrates for peroxidation and are oxidized through the coordinated actions of lipid metabolism-related enzymes such as acyl-CoA synthetase long-chain family member 4 (ACSL4), lysophosphatidylcholine acyltransferase 3 (LPCAT3), and arachidonate lipoxygenase (ALOX) family members (Kagan et al., 2017; Doll et al., 2017). On the other hand, inhibition of the System Xc^−^–GSH–GPX4 pathway impairs lipid peroxide detoxification, leading to membrane damage and ferroptotic cell death. Due to dysregulated iron metabolism, increased lipid metabolic activity and oxidative stress, modulation of ferroptosis not only serves as a critical mediator of the fate of tumor cells in digestive system malignancies but is also a potential therapeutic target.

The biological consequences of ferroptosis in digestive system tumors are highly context dependent. In CRC, ferroptosis has been linked to tumor proliferation, immune regulation, therapeutic resistance, and metastatic progression (Wang et al., 2019). Sorafenib, an agent used in HCC, also exerts antitumor activity by inducing ferroptosis, whereas nuclear factor erythroid 2-related factor 2 (NRF2) and GPX4 are activated as therapeutic resistance factors via antioxidant pathways (Liu et al., 2022). The solute carrier family 7 member 11 (SLC7A11), GPX4, PI3K/AKT and NRF2 signaling axes maintain antioxidant capacity and promote ferroptosis resistance in GC. Kirsten rat sarcoma viral oncogene homolog (KRAS)--dependent metabolic reprogramming influences ferroptosis sensitivity in pancreatic cancer by reshaping lipid metabolism, iron handling, and redox homeostasis (Xu et al., 2024). Transcriptional and epigenetic regulation, lactate and amino acid metabolism, exosome-mediated intercellular communication, immune cytokine signaling and reciprocal TME feedback can also reshape ferroptotic responses to form a multilayered regulatory network.

These mechanistic advances have stimulated interest in therapeutic strategies that target ferroptosis in digestive system cancers. Ferroptosis inducers can promote tumor-cell death by inhibiting the System Xc^−^–GSH–GPX4 axis, increasing intracellular iron availability, enhancing ROS production, or disrupting metabolic and redox homeostasis (Qiao et al., 2024). Further improvement of tumor selectivity, microenvironment-responsive drug release and amplification of Fenton reactions and oxidative damage can be achieved by the use of nanomaterial-based platforms and drug delivery systems. In addition, multimodal therapeutic strategies combining ferroptosis induction with immunotherapy, and potentially with chemotherapy or targeted therapy, offer a promising opportunity to enhance antitumor efficacy and overcome the limitations of monotherapy (Cheng et al., 2024). Nonetheless, the clinical translation of these candidates is restricted by complicated ferroptosis regulatory networks, significant intratumoral heterogeneity, context-dependent resistance and limited available predictive biomarkers (Wa et al., 2022). In recent studies, ferroptosis-related mechanisms and therapeutic resistance in digestive system cancers have been increasingly investigated (Yang et al., 2023). However, several important issues, including cancer-specific vulnerabilities, context-dependent responses, and translational challenges, remain incompletely understood. Nonetheless, these complementary views still fail to comprehensively integrate several critical issues, including how to distinguish shared ferroptotic vulnerabilities from cancer-specific escape programs across major digestive system malignancies, how to interpret context-dependent and seemingly contradictory therapeutic effects observed in different experimental settings, and how to reconcile mechanistic discoveries with emerging clinical translational evidence. A critical comparison of recent reviews and the residual analytical gap addressed in the present Review is provided in Table 1. This Review builds on these issues with a collective iron–lipid–redox vulnerability axis trained from the mechanistic-to-translational literature that connects unique cancer resistance architectures and evidence-informed, biomarker-guided therapeutic stratification components through a ferroptosis-centric lens. One such representative example comes from BRAF^V600E^ CRC, where the PLK1–CBX8–GPX4 signaling axis governs ferroptosis-dependent therapeutic resistance in a setting of non-MAPK dependent regulation, serving as an example of how tumor-specific regulatory networks dictate responses to ferroptosis (Zhao Z. et al., 2025). Given the comparatively extensive and mechanistically developed evidence currently available, the cancer-type-specific discussion therefore primarily focuses on CRC, HCC, GC, and PDAC. The scope of this Review was intentionally restricted to four major digestive system malignancies: CRC, HCC, GC, and PDAC. This selection was based on the availability of mechanistically validated ferroptosis studies rather than disease prevalence alone. These four cancer types provide relatively comprehensive evidence covering iron metabolism, lipid remodeling, antioxidant regulation, TME interactions, and ferroptosis-targeted therapeutic strategies. Other digestive system malignancies were not excluded because ferroptosis is irrelevant; rather, current evidence in some areas remains comparatively limited, as exemplified by the still-unclear regulatory role of HMGA1 in ferroptosis within EC, and is therefore presented as emerging evidence instead of independent cancer-specific sections (Yang J-Y. et al., 2024). Other selected studies related to various other digestive system neoplasms, especially esophageal squamous cell carcinoma (ESCC) and extrahepatic cholangiocarcinoma, are also included as extensions of evidence to demonstrate the broader universality and tumor-type-dependent specificity in how ferroptosis is regulated. The rest of this chapter thus seeks to address these additional, i.e., non-core cancer types in so far as they do not receive the same depth of discussion as those four core cancer types.

Thus, the synthesis is structured on three *a priori* questions: (1) What are the shared susceptibilities in CRC, HCC, GC and PDAC with respect to iron–lipid–redox ferroptosis machinery components; conversely, what resistance programs are specific for each cancer type? (2) What tumor-intrinsic and microenvironmental determinants underlie the observed context-dependence of ferroptosis induction yielding disparate or contrasting therapeutic outcomes? (3) What candidate biomarkers and therapeutic combinations have only mechanistic or preclinical evidence of validity, and what validation steps are required before patient stratification/clinical decision-making based on these parameters?

To address these questions, this Review was conducted as a formal narrative review. The search strategy, study selection criteria, and approaches for evidence extraction and synthesis are outlined in Section 2. Evidence was classified according to translational maturity and biological context, while accounting for multiple contextual variables, including tumor type, cellular compartment, treatment setting, experimental model and microenvironmental conditions, as well as the level of available evidence. Thus, the synthesis framework and biomarker-guided model developed in this Review are intended to generate testable hypotheses rather than represent a clinically validated predictive classification of tumors.

## 2 Literature search and evidence synthesis

### 2.1 Review design and literature search

This review was conducted as a structured narrative review designed to comparatively synthesize mechanistic and translational evidence on ferroptosis in digestive system malignancies rather than as a systematic review or meta-analysis. Literature searches were conducted in PubMed/MEDLINE, Web of Science Core Collection, and Scopus from database inception to 30 August 2026. The search combined terms related to ferroptosis with terms for digestive system malignancies. The core search concept was: (“ferroptosis” OR “ferroptotic”) AND (“CRC” OR “colon cancer” OR “rectal cancer” OR “GC” OR “stomach cancer” OR “HCC” OR “liver cancer” OR “pancreatic cancer” OR “PDAC” OR “esophageal cancer” OR “cholangiocarcinoma” OR “biliary tract cancer”). Search syntax was adapted to the requirements of each database. Reference lists of key primary studies and recent reviews were additionally screened to identify relevant studies not captured by the electronic search.

### 2.2 Eligibility and study selection

English-language peer-reviewed studies were considered eligible when they directly examined ferroptosis-related mechanisms, regulatory networks, therapeutic resistance, tumor–microenvironment interactions, biomarkers, or ferroptosis-targeted interventions in digestive system malignancies. Primary mechanistic studies, animal studies, patient-derived models, human multi-omics or spatial studies, and clinically relevant studies were eligible. Recent reviews were included primarily to define the existing review landscape and identify residual analytical gaps. Studies were excluded when ferroptosis was mentioned only indirectly without mechanistic or functional relevance, when the disease context was unrelated to digestive system malignancies, or when sufficient methodological information was unavailable. For cancer-type-specific synthesis, malignancies were prioritized when sufficient mechanistic evidence was available to evaluate ferroptosis regulation across multiple biological dimensions, including iron metabolism, lipid metabolism, antioxidant dependency, TME regulation, and therapeutic intervention. Accordingly, CRC, HCC, GC, and PDAC were selected as the four core digestive system malignancies for detailed comparative analysis, whereas other tumor types with relatively limited evidence were considered as emerging evidence.

Titles and abstracts were independently screened by Xiaoguang Qin and Qingna Wen, followed by full-text assessment of potentially relevant articles. Disagreements regarding study eligibility or interpretation were resolved through discussion and, when consensus could not be reached, adjudicated by Kaikai Wang.

### 2.3 Data extraction, claim-level source verification, and evidence synthesis

For each included primary study, two authors (Xiaoguang Qin and Qingna Wen) independently extracted full-text information on cancer type, experimental model or patient population, intervention, ferroptosis-related target or pathway, direction of ferroptosis regulation, validation criteria, major biological or therapeutic endpoints, treatment sensitivity or resistance, translational evidence level, and principal limitations.

All mechanistic statements in the Review were independently cross-checked against the full text of the cited primary studies to confirm consistency in cancer type, affected cell type, experimental model, molecular mechanism, direction of ferroptosis regulation, biological outcome, and evidence strength. These parameters were also used to guide evidence classification and the comparative presentation of figures and tables throughout the Review. Ferroptosis-specific evidence was evaluated according to reported experimental criteria, including lipid peroxidation or lipid ROS, intracellular Fe^2+^ changes, GSH depletion, GPX4/SLC7A11 alterations, ferroptosis-associated morphological changes, and pharmacological or genetic rescue where available, as exemplified by the use of C11-BODIPY staining, FerroOrange staining, GSH ratio measurement, malondialdehyde (MDA) quantification, and transmission electron microscopy to assess ferroptosis (Lin H. et al., 2025). Claims lacking direct support or relying only on nonspecific ROS or cell-viability changes were revised, restricted, reassigned, removed, or interpreted cautiously.

For evidence attribution, ferroptosis was considered to be causally supported when lipid-peroxidation-associated cell death was accompanied by ferroptosis-specific pharmacological rescue and/or genetic manipulation of core ferroptosis machinery where available, as exemplified by the restoration of the inhibitory effects of LGX818 ± cetuximab plus ras-selective lethal 3 (RSL3) by the ferroptosis inhibitors Fer-1 and Lip-1 in HT-29 and WiDr cells (Zhao Z. et al., 2025). Changes limited to ROS, MDA, GSH, GPX4/SLC7A11 expression, or cell viability without functional rescue were classified as supportive rather than definitive evidence of ferroptosis. When multiple cell-death programs were simultaneously activated, the contribution of ferroptosis was interpreted separately from apoptosis, pyroptosis, cuproptosis, or other death modalities unless direct causal discrimination was experimentally demonstrated.

Disagreements were resolved through discussion and, when necessary, adjudicated by Kaikai Wang. The extracted study-level information was incorporated into the evidence-graded summaries in Table 2 and Supplementary Table 1 to facilitate claim-to-source verification and comparison across cancer types, experimental models, therapeutic interventions, and levels of translational evidence. To ensure consistent interpretation of translational evidence, each therapeutic intervention was additionally annotated according to cancer type, experimental model, comparator, ferroptosis-validation criteria, treatment effect direction, safety assessment, and independent replication status. Evidence was classified according to a predefined translational hierarchy: (i) biochemical or cell-line evidence; (ii) organoid or patient-derived *ex vivo* model evidence; (iii) *in vivo* animal model evidence; (iv) patient-derived xenograft (PDX) model evidence; (v) retrospective human tissue or clinical association evidence; (vi) prospective biomarker study evidence; (vii) interventional clinical trial evidence; and (viii) approved clinical indication evidence. Throughout this Review, mechanistic findings, preclinical therapeutic evaluation, human association studies, and clinically validated therapeutic efficacy were considered distinct evidence categories and were not interpreted interchangeably.

**TABLE 1:** Critical comparison of recent reviews and the residual analytical gap addressed in the present Review.

Review	Publication date	Cancer/disease coverage	Evidence emphasized	Central analytical lens	Distinctive focus and residual gap relevant to the present review
Rabitha et al. (2023)	2023	GI tumors	Mainly mechanistic/preclinical	Ferroptosis pathways and therapeutic strategies	Broad pathway-to-therapy synthesis; does not explicitly distinguish shared vulnerabilities from cancer-specific resistance architecture
Quan et al. (2024)	2024	Digestive system tumors	Mechanistic/preclinical	Ferroptosis and treatment tolerance	Focuses on treatment tolerance rather than integrating cross-cancer vulnerability, resistance heterogeneity and biomarker-guided translation
Escuder-Rodriguez et al. (2024)	2024	GI diseases including cancer	Mechanistic/disease biology	General ferroptosis biology in GI disease	Broad disease scope; not organized around comparative cancer-specific resistance states or patient stratification
Chen et al. (2025b)	2025/2026	Digestive cancers	Mechanistic/preclinical	Ferroptosis-mediated reversal of drug resistance, including tumor heterogeneity, metabolic alterations, tumor–stroma interactions, and TME-associated resistance mechanisms	Provides a focused synthesis of ferroptosis in overcoming therapeutic resistance across digestive cancers. The present Review extends this perspective by comparatively separating shared ferroptotic vulnerability from cancer-specific escape programs and by explicitly distinguishing mechanistic, *in vivo*, patient-derived, and clinical levels of evidence for biomarker-guided translation
Zhang et al. (2025g)	2025	GI cancers	Mechanistic/preclinical	Integrated ferroptosis–cuproptosis mechanisms, metabolic vulnerabilities, therapeutic interventions, biomarkers, and translational pathways in gastrointestinal cancers	Provides a broad metal-dependent cell-death framework that integrates ferroptosis and cuproptosis and includes molecular subtyping and biomarker-oriented translation. The present Review differs by adopting a ferroptosis-specific cross-cancer comparative framework that explicitly links a shared iron–lipid–redox vulnerability axis to cancer-specific resistance programs and stratifies the supporting evidence according to translational maturity
Guo et al. (2026b)	2026	Digestive tract tumors	Mechanistic/TME/immune	Dual roles of ferroptosis with emphasis on TME regulation, immune interactions, combination therapy, and biomarker-oriented clinical translation	Provides a detailed account of the bidirectional ferroptosis–TME–immune relationship and highlights multidimensional biomarkers for patient stratification. The present Review adopts a complementary cross-cancer analytical structure that separates shared ferroptotic vulnerability from tumor-specific resistance programs and explicitly maps candidate biomarkers and therapeutic strategies to different levels of translational evidence
Present Review	​	CRC, HCC, GC, PDAC as core cancers; selected ESCC/cholangiocarcinoma	Mechanistic, *in vivo*, patient-derived, human multi-omics/spatial, and clinical evidence explicitly distinguished	Ferroptosis-specific cross-cancer synthesis: shared iron–lipid–redox vulnerability → cancer-specific resistance architecture → evidence-informed biomarker and therapeutic matching	The proposed vulnerability/resistance states remain hypothesis-generating and require prospective validation using prespecified biomarkers, quantitative thresholds, and independent patient cohorts

**TABLE 2:** Overview of the differential roles of ferroptosis and intervention strategies in various digestive system tumors.

Tumor type	Ferroptosis-related features	Key regulatory molecules/signaling pathways	Major biological effects	Impact on drug sensitivity/resistance	Potential therapeutic strategies	Evidence level/model system	References
CRC	USP8 inhibition promotes lipid peroxidation and enhances ferroptosis sensitivity; USP8 suppresses ferroptosis by stabilizing GPX4	USP8–GPX4 axis; System Xc^−^–GSH–GPX4 pathway	Inhibits tumor growth; promotes CD8^+^ T cell infiltration; enhances antitumor immunity	High USP8 expression confers ferroptosis resistance; USP8 inhibition increases sensitivity to ferroptosis inducers and immunotherapy	USP8 inhibitors (e.g., DUB-IN-2) combined with ferroptosis inducers, including sulfasalazine (SAS) and imidazole ketone erastin (IKE), and anti–PD-1 immunotherapy	Preclinical evidence; HCT116/SW620 CRC cell lines, IEC-specific Usp8 knockout mice, AOM/DSS-induced CRC model, and CT26 syngeneic tumor model	(Li et al., 2024a)
PDAC	CAF-derived exosomes suppress ferroptosis by inhibiting ACSL4 and reducing lipid ROS levels	CAFs–exosomes–miR-3173-5p–ACSL4 axis	Promotes tumor cell survival and maintains tumor–stroma signaling communication	Inhibition of ferroptosis enhances resistance to GEM	Targeting CAF-derived exosomes or the miR-3173-5p/ACSL4 axis restores ferroptosis sensitivity	Preclinical evidence; primary human PDAC CAF/NF-derived exosomes, PANC-1 and BxPC-3 cell models, TCGA-PDAC clinical dataset, and PANC-1 xenograft mouse model	(Qi et al., 2023)
GC	STAT3 suppresses lipid peroxidation and ferroptosis by upregulating GPX4, SLC7A11, and ferritin heavy chain 1 (FTH1); inhibition of STAT3 induces ferroptosis (increased lipid ROS and Fe^2+^)	STAT3–GPX4/SLC7A11/FTH1 ferroptosis negative regulatory axis (FNR)	Promotes tumor growth, progression, and cell survival; inhibition of STAT3 suppresses tumor growth	STAT3 activation and upregulation of FNR genes confer resistance to 5-FU; induction of ferroptosis restores chemosensitivity	STAT3 inhibitor (W1131) induces ferroptosis; combination with 5-FU or ferroptosis inducers (such as erastin) overcomes resistance	Preclinical evidence; AGS/MGC803/HGC27 GC cell models, 5-FU-resistant MGC803/5-FU cells, patient tissue and TCGA/GEO analyses, MGC803 xenografts, GC PDX and PDX-derived organoid models	(Ouyang et al., 2022)
Metastatic gastric adenocarcinoma (GAC)	Upregulation of GPX4 during tumor progression leads to ferroptosis evasion	GPX4-mediated ferroptosis resistance pathway	Promotes tumor growth and metastatic progression; tumor cells co-evolve with the microenvironment	Ferroptosis resistance reduces the efficacy of CAR-T immunotherapy; restoration of ferroptosis enhances immunotherapeutic response	Inhibition of GPX4 or restoration of ferroptosis sensitivity combined with CAR-T therapy	Translational/preclinical evidence; paired primary–metastatic GAC patient samples with single-cell profiling, GAC cell lines, PDX models, KPCS; Gpx4 conditional knockout mouse model, and metastatic xenograft models	(Cheng et al., 2024)
GC	Hypoxia inhibits ferroptosis by suppressing lipid peroxidation and iron-dependent reactions (↓ROS, ↓MDA, ↓Fe^2+^ activity)	Spliced X-box binding protein 1 (XBP1s)–myeloid-derived growth factor (MYDGF)–UBQLN1–lipocalin 2 (LCN2) axis (LCN2 stabilization → iron chelation → inhibition of ferroptosis)	Promotes tumor cell survival and adaptation under hypoxic conditions	Inhibition of ferroptosis reduces sensitivity to ferroptosis inducers (e.g., IKE); disruption of this axis enhances therapeutic efficacy	Targeting XBP1s/MYDGF or disrupting LCN2 stability in combination with ferroptosis inducers	Preclinical mechanistic evidence; GC scRNA-seq datasets, AGS and HGC27 cell models under hypoxia, MYDGF/XBP1s/LCN2 genetic manipulation, and HGC27 xenograft mouse model	(Zhou et al., 2026)
GC	High expression of GPX4 and SLC7A11 inhibits ferroptosis; combination therapy promotes lipid peroxidation (↑ROS, ↑MDA, ↓GSH) to induce ferroptosis	SLC7A11/GSH/GPX4 axis; PI3K/AKT/mTOR signaling pathway	Induces ferroptosis and inhibits tumor growth and metastasis	Apatinib monotherapy is associated with resistance and toxicity; combination with Chi-Ag enhances drug sensitivity and overcomes resistance	Apatinib combined with silver nanoparticles (HA-Ag-PLGA@Apa) delivery system to target and induce ferroptosis	Preclinical evidence; GC cell lines (BGC-823, HGC-27, SGC-7901), 3D BGC-823 spheroids, human GC tissue validation, and BGC-823 xenograft mouse model	(Lin et al., 2025b)
GAC	Ornithine decarboxylase 1 (ODC1) inhibits ferroptosis by suppressing lipid peroxidation and reducing GSH consumption	ODC1–Y-box-binding protein 1 (YBX1)–SLC7A11 transcriptional regulatory axis	Promotes cell proliferation, migration, invasion, and tumor growth	Upregulation of ODC1 leads to 5-FU resistance; inhibition of ODC1 or induction of ferroptosis restores chemosensitivity	Targeting ODC1 or YBX1 to suppress SLC7A11 expression; combination of ferroptosis inducers (e.g., erastin) with 5-FU therapy	Preclinical mechanistic evidence with human correlative validation; STAD patient tissues and transcriptomic/scRNA-seq datasets, AGS/HGC27/MKN45 and 5-Fu-resistant Re-AGS/Re-HGC27 cell models, and nude-mouse xenograft models	(Li et al., 2026b)
CRC	The NheA-O complex induces lipid peroxidation (↑ACSL4, ↓GPX4), triggering ferroptosis-like cell death	β-catenin–GPX4 axis; GPX4 proteasomal degradation mechanism	Inhibits cell proliferation, migration, colony formation, and ATP production; induces tumor cell death	Synergizes with the ferroptosis inducer RSL3 to enhance cell death; the ferroptosis inhibitor Fer-1 partially reverses this effect	Bacterial protein–oleic acid complex (NheA-O) as a novel ferroptosis inducer; combination with RSL3 enhances antitumor effects	Preclinical *in vitro* evidence; DLD1/HCT8 CRC cell models and DLD1 3D spheroids, with supportive TCGA analysis; no *in vivo* validation	(Ullah et al., 2026)
CRC	DHA induces lipid peroxidation and oxidative stress, triggering ferroptosis	Lipid peroxidation-driven mechanism (PUFA metabolism-associated oxidative stress)	Inhibits tumor cell viability and eliminates PDOs	Enhances the efficacy of oxaliplatin; suppresses regeneration of drug-tolerant persister cells	Utilization of DHA as a low-toxicity ferroptosis inducer in combination with chemotherapy	Preclinical *in vitro* evidence; HT29 CRC cell model and patient-derived CRC tumor organoids (PDTOs), including oxaliplatin-tolerant persister cell models; no *in vivo* validation	(di Blasio et al., 2026)
CRC	Activation of HIF-1α suppresses lipid peroxidation and promotes resistance to ferroptosis; its inhibition enhances ferroptosis (↑ROS, ↑MDA, ↓GSH)	HIF-1α–P4HA1 transcriptional regulatory axis; hypoxia signaling pathway	Promotes tumor growth, metastasis, and formation of an immunosuppressive microenvironment	Upregulation of HIF-1α leads to resistance to ferroptosis inducers (e.g., RSL3) and reduced sensitivity to anti-PD-1 immunotherapy; inhibition of HIF-1α restores sensitivity	HIF-1α inhibitors (e.g., BAY 87–2,243) in combination with ferroptosis inducers (RSL3) and anti-PD-1 immunotherapy	Preclinical mechanistic evidence; MSS CRC cell models (SW480, HT-29, WiDr), RSL3-resistant Re-CT26 cells, and BALB/c CT26 syngeneic subcutaneous/liver-metastasis models, with scRNA-seq and patient-dataset analyses	(Yang et al., 2026)
CRC	Copper/iron ion overload induces oxidative stress and lipid peroxidation, triggering ferroptosis	Metal ion metabolism-related pathways (Cu^2+^/Fe^2+^ overload) and oxidative stress mechanisms	Induces ferroptosis, cuproptosis, and pyroptosis, inhibits tumor growth, and activates antitumor immunity	Enhances responsiveness to immune checkpoint blockade and strengthens antitumor immune effects	Laser-activated nanoplatform (CuFeS2-CA-DAC-Lipo, CCDL) in combination with immunotherapy	Preclinical evidence; CRC cell models and *in vivo* tumor models with immune-microenvironment and ICB-response validation; no clinical evidence	(Zhao et al., 2026)
ESCC	Upregulation of Zrt- and Irt-like Protein 8 (ZIP8) inhibits ferroptosis (↑GPX4, ↓lipid peroxidation); ZIP8 knockdown promotes ferroptosis (↑Fe^2+^, ↑lipid peroxidation)	ZIP8–Zn^2+^–pCREB–GPX4 signaling axis	Promotes tumor cell proliferation and tumor growth	ZIP8 overexpression reduces sensitivity to the ferroptosis inducer erastin; inhibition of ZIP8 enhances sensitivity to erastin	nobiletin targets ZIP8 to suppress its expression and induce ferroptosis	Preclinical/translational evidence; ESCC patient tissues and TCGA/GEO analyses, KYSE-series ESCC cell models, and Nobiletin-treated PDX mouse models	(Yang et al., 2025g)
Extrahepatic cholangiocarcinoma	Upregulation of AKR1C1 inhibits ferroptosis (↓lipid ROS, ↓Fe^2+^); aldo-keto reductase family 1 member C1 (AKR1C1) knockdown promotes ferroptosis (↑lipid peroxidation, ↑iron levels)	AKR1C1–CYP1B1–cAMP–PKA signaling axis (AHR involved in transcriptional regulation)	Promotes tumor cell proliferation, tumor growth, and poor prognosis	Loss of AKR1C1 enhances sensitivity to ferroptosis inducers (erastin, RSL3)	Targeting AKR1C1 (gene knockdown or inhibitors) in combination with ferroptosis inducers (e.g., IKE)	Translational/preclinical evidence; human ECC tissues, QBC939 cell models with inducible AKR1C1 perturbation, and BALB/c nude-mouse QBC939 xenografts with ferroptosis-inducer validation	(Liu et al., 2025a)
HCC	SLC12A5 upregulates xCT to promote GSH synthesis and reduce lipid peroxidation, thereby inhibiting ferroptosis	SLC12A5–ER stress–Ca^2+^–PNCK–PI3K/AKT/mTOR signaling axis; xCT (SLC7A11)-mediated cystine transport	Promotes tumor cell proliferation, cell cycle progression, and tumor growth	Inhibition of SLC12A5 or use of its antagonists enhances ferroptosis	SLC12A5 inhibitor (VU0240551) induces ferroptosis and inhibits tumor growth	Translational/preclinical evidence; human HCC tissues, Huh7/HepG2 cell models, Huh7 xenografts, and VU0240551-treated HCC PDX models	(Tong et al., 2023b)
Pancreatic cancer (KRAS-mutant)	Tropomodulin 3 (TMOD3) inhibits ferroptosis by promoting ACSL4 protein degradation	KRAS–ELK1–TMOD3–F-actin–autophagosome–lysosome fusion–ACSL4 axis	Promotes tumor immune evasion and suppresses antitumor immune responses	Upregulation of TMOD3 leads to resistance to ferroptosis inducers and anti-PD-1 immunotherapy; inhibition of TMOD3 restores sensitivity	Targeting TMOD3 (e.g., cangrelor) in combination with PD-1 antibodies enhances immunotherapeutic efficacy	Preclinical mechanistic evidence with human tissue validation; KRAS-mutant/wild-type PC cell models, human PC tissues, and mouse tumor models with TMOD3-targeting/anti-PD-1 validation	(He et al., 2025)

The cancer-type-specific discussion in the main text primarily focuses on CRC, HCC, GC, and PDAC, because these malignancies currently have comparatively extensive mechanistic and therapeutic evidence regarding ferroptosis. Selected studies involving other digestive system malignancies, including ESCC, and extrahepatic cholangiocarcinoma, are included as expanded evidence to illustrate the broader applicability and tumor-type-specific heterogeneity of ferroptosis-related regulatory mechanisms. Their inclusion does not imply an equivalent level of evidence or depth of discussion to the four core cancer types.

Because the included literature spans heterogeneous experimental designs, a formal meta-analytic risk-of-bias score was not applied. Instead, evidence was qualitatively appraised according to model relevance, direct experimental support for ferroptosis, availability of *in vivo* or patient-derived validation, consistency with independent studies, and translational proximity.

Conflicting or negative findings were retained when directly relevant to the review questions. Rather than resolving discrepancies by simple vote counting, apparently inconsistent findings were interpreted according to tumor type, cellular compartment, treatment context, experimental model, microenvironmental conditions, and level of evidence. Findings based only on correlative associations or single-model observations were interpreted more cautiously than those supported by functional validation across multiple experimental levels.

## 3 Molecular basis of ferroptosis

The molecular basis of ferroptosis is the common vulnerability layer under the proposed framework. Iron availability, the abundance and peroxidizability of membrane lipids, and the capacity of cellular antioxidant systems should be envisioned as components on a system-wide iron–lipid–redox axis instead of independent pathways. This three-component balance defines the basal ferroptotic threshold where tumor-specific regulatory networks act.

### 3.1 Dysregulated iron metabolism

Dysregulated iron homeostasis is a central molecular determinant of ferroptosis and provides a mechanistic basis for understanding ferroptosis susceptibility in digestive system malignancies. Iron metabolism is further influenced by metabolic stress within the TME, and the resulting changes can affect both ferroptotic sensitivity and therapeutic resistance, as summarized in Figure 1. In a physiological context, intracellular iron homeostasis is carefully controlled by the concerted actions of iron uptake, transport, storage, utilization and export to sustain mitochondrial respiration, DNA synthesis and multiple enzymatic reactions. Here, the mechanisms leading to ferroptosis are discussed, which involve various cellular programs such as dysregulation of iron homeostasis (Brown et al., 2024). When iron status is disrupted, the labile iron pool increases, mostly due to Fe^2+^ accumulation that enhances ROS production by the Fenton reaction and promotes membrane lipid peroxidation, inducing lipid peroxide formation and cell death by ferroptosis (Caneque et al., 2025). Thus, expansion of the labile iron pool contributes to ferroptotic susceptibility by increasing redox-active iron and oxidative pressure; however, its effect is contingent on the availability of peroxidizable membrane phospholipids and the capacity of cellular antioxidant defenses.

**FIGURE 1:**
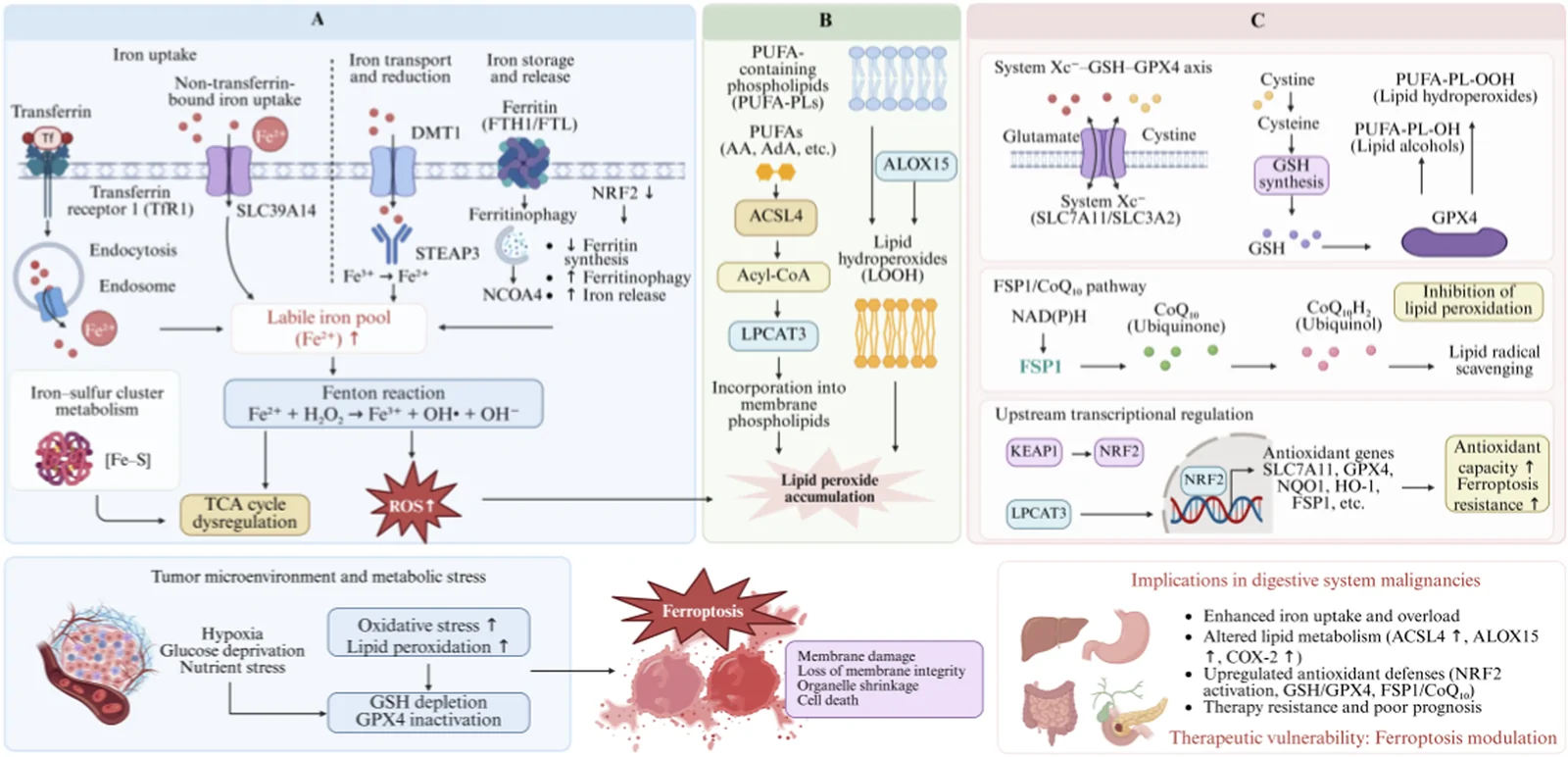
Core iron–lipid–redox determinants of ferroptosis vulnerability in digestive system malignancies. Uptake, trafficking and degradation of iron and ferritinophagy expand the labile Fe^2+^ pool. An overload of Fe^2+^ promotes the Fenton reaction, resulting in ROS production, cellular oxidative stress and at last lipid peroxidation. PUFA substrates associated with ACSL4, such as AA and AdA, are incorporated into phospholipids via LPCAT3. Lastly, ALOX15-generated phospholipid hydroperoxides and ROS-mediated oxidation initiate dosage of lipid peroxide in addition to plasma membrane damage and ferroptosis. The synergistic action involves phospholipid hydroperoxide elimination by the System Xc^−^–GSH–GPX4 axis and lipid radical propagation restriction via the FSP1/CoQ10 pathway. KEAP1–NRF2 signaling has also enhanced expression of pro-survival and antioxidant genes including SLC7A11, GPX4, NQO1 and HO-1. Ferroptosis promoted by hypoxia, glucose deprivation and nutrient starvation induces oxidative stress, GSH depletion and GPX4 inactivation. Together, iron homeostasis, lipid metabolic reprogramming and antioxidant capacity determine ferroptosis susceptibility and affect the resistance to cutting-edge therapy as well as the response to a promising treatment strategy targeting ferroptosis in digestive system malignancies. Abbreviations: AA, arachidonic acid; ACSL4, acyl-CoA synthetase long-chain family member 4; AdA, adrenic acid; CoQ10, coenzyme Q10; FSP1, ferroptosis suppressor protein 1; GPX4, glutathione peroxidase 4; GSH, glutathione; KEAP1, Kelch-like ECH-associated protein 1; LPCAT3, lysophosphatidylcholine acyltransferase 3; NRF2, nuclear factor erythroid 2-related factor 2; PUFAs, polyunsaturated fatty acids; ROS, reactive oxygen species; SLC7A11, solute carrier family 7 member 11; System Xc^−^: cystine/glutamate antiporter system. **(A)** Dysregulated iron metabolism. **(B)** Lipid peroxidation. **(C)** Antioxidant systems.

**FIGURE 2:**
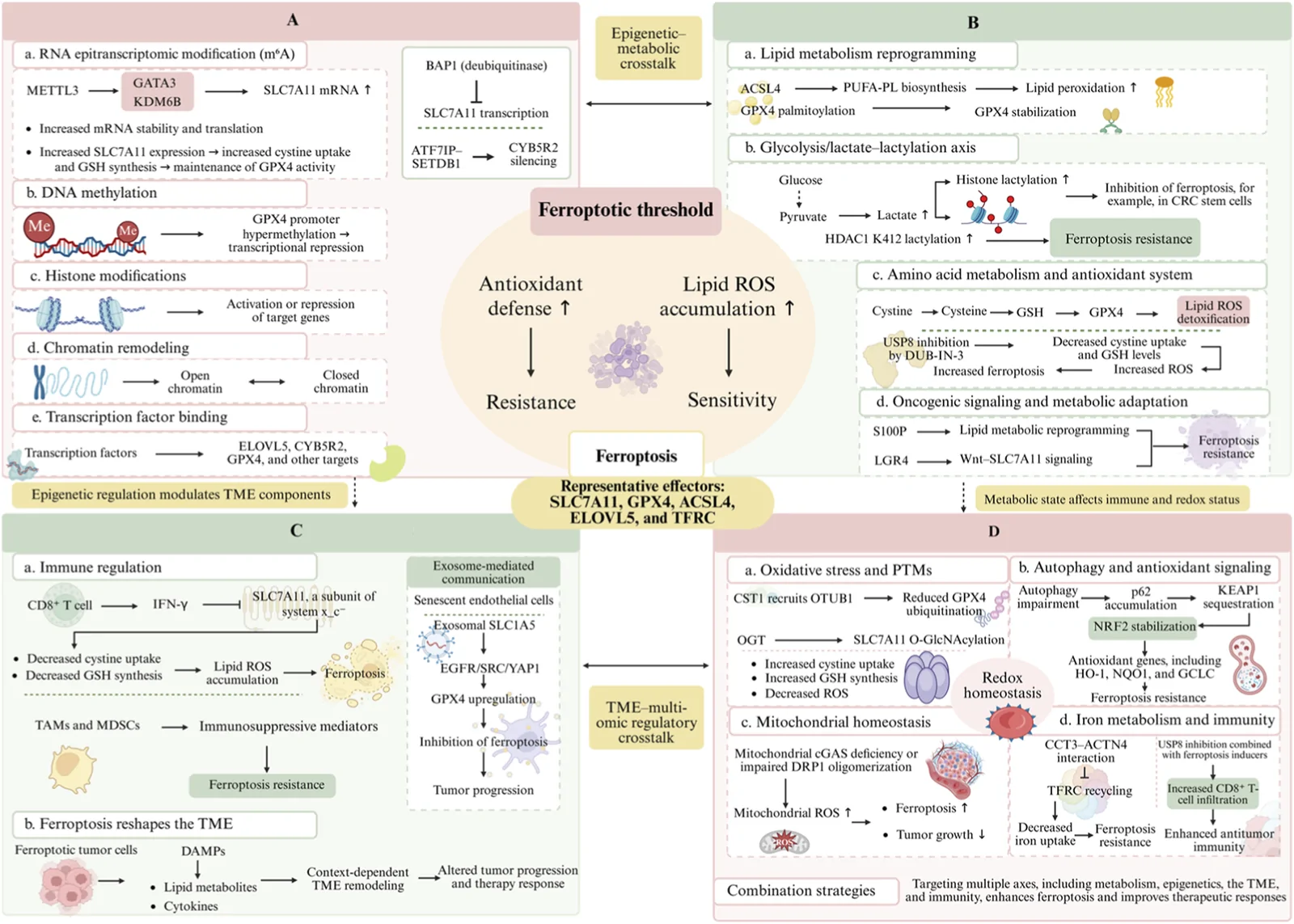
Multilevel regulatory modifiers of the iron–lipid–redox ferroptosis vulnerability axis. Regulation of ferroptosis is achieved via transcriptional and epigenetic mechanisms affecting key ferroptosis-related genes such as SLC7A11, GPX4, ELOVL5 and CYB5R2 through RNA modifications, DNA methylation, histone modifications, chromatin remodelling and transcription-factor binding. The ferroptosis response is mediated by metabolic reprogramming via PUFA-phospholipid biosynthesis, GPX4 stabilization, glycolysis–lactate–lactylation axis; cysteine-GSH metabolism and oncogenic metabolic adaptation. Augmented susceptibility to ferroptosis is mediated by TME-derived signals and exosome-mediated intercellular communication that reshape redox homeostasis, metabolism, and immune signaling. Conversely, ferroptotic tumor cells can release damage-related signals that rewire the TME and influence tumor progression and therapeutic response. The interplay between oxidative stress, post-translational modifications, autophagy–NRF2 signaling, mitochondrial homeostasis, iron metabolism and antitumor immunity resonate to determines the cellular redox balance. These interlinked mechanisms govern the equilibrium of antioxidant capacity and lipid ROS formation, thus governing ferroptosis susceptibility or resistance and resultant responses to combination therapies in these malignancies (CRC, GC, HCC, PDAC). Arrows indicate activation or promotion and blunt-ended lines indicate inhibition. Abbreviations: CRC, colorectal cancer; ELOVL5, elongation of very long-chain fatty acids protein 5; GC, gastric cancer; GPX4, glutathione peroxidase 4; GSH, glutathione; HCC, hepatocellular carcinoma; NRF2, nuclear factor erythroid 2-related factor 2; PDAC, pancreatic ductal adenocarcinoma; PUFA, polyunsaturated fatty acid; ROS, reactive oxygen species; SLC7A11, solute carrier family 7 member 11; TME, tumor microenvironment. Validated mechanisms supported by functional studies are distinguished from regulatory associations and emerging hypotheses. Cancer-specific examples and evidence levels are indicated where available. Mechanisms supported by functional validation are distinguished from associative observations and emerging hypotheses according to available evidence levels. Cancer-specific examples and experimental contexts are indicated where applicable. **(A)** Transcriptional and epigenetic regulation. **(B)** Metabolic reprogramming. **(C)** TME regulation of ferroptosis. **(D)** Interplay among regulatory mechanisms.

Excess iron absorption constitutes one of the main mechanisms contributing to intracellular iron overload. Transferrin-dependent endocytosis is the classical route for the acquisition of iron and SLC39A14 mediates non-transferrin-bound iron (NTBI) uptake; in hepatocyte models of liver-graft ischemia-reperfusion injury, SLC39A14 knockdown reduces NTBI uptake, iron overload, and ferroptosis, supporting a direct link between this transporter and ferroptotic iron loading (Deng Z. et al., 2025). In addition to iron uptake, dysregulated iron transport and reduction pathways also promote the relative expansion of the reactive iron pool. Increased intra-cellular transport of iron by divalent metal transporter 1 and enhanced STEAP3-mediated conversion of Fe^3+^ into Fe^2+^ result in a greater proportion of redox-active iron. At the same time, changes in ferritin-based iron storage lead to iron accumulation and free iron release which exacerbate the susceptibility of cells to ferroptosis (Yang J. et al., 2025). Disordered iron homeostasis also largely results from dysregulated iron storage and utilization. Ferritin is the principal cellular iron-storage complex, which sequesters excess iron into a relatively inert state, limiting free and highly reactive Fe^2+^ from participating in the Fenton reaction that leads to toxicity. Moreover, neutral cytosolic pH further limits iron bioavailability and balances Fenton chemistry by keeping iron sequestered within the protein ferritin (Li S. et al., 2026). In contrast, dysfunctional ferritin may cause elevated levels of reactive iron within a cell or misdirected lipid peroxidation, boosted by the gain-of-function of transferrin receptor or reduced degradation and iron release from excess ferritin. Loss of NFE2L2/NRF2, for instance, alters the expression of crucial regulators of ferritin synthesis and its turnover, perturbing the delicate balance in iron homoeostasis and expanding the labile iron pool, thereby rendering tumor cells susceptible to ferroptosis (Anandhan et al., 2023).

In addition to classic iron uptake and storage pathways, alterations in iron–sulfur cluster metabolism and the action of iron chaperone proteins have increasingly been identified as mediators of iron dyshomeostasis. CRC cells in digestive system malignancies can modify intracellular iron distribution and free iron availability via dysregulated expression of iron chaperones. For instance, Acevaltrate (ACE) acts as a dual-target ferroptosis inducer in CRC by downregulating the iron chaperones poly(rC)-binding protein (PCBP) 1/2, thereby increasing intracellular Fe^2+^, while also directly targeting GPX4 to weaken lipid-peroxide detoxification and promote ferroptosis (Yu et al., 2025).

All told, the iron dysregulation in digestive system tumors appears to be representative of a closely coordinated rewiring of multiple pathways mediating iron uptake, transport and reduction, storage, or iron–sulfur cluster biogenesis, whether mediated by altered expression or loss-of-function mutations of genes involved in these pathways or possibly even cooperatively acting changes (Ciscar et al., 2026). Taken together, these changes reinforce an intracellular labile iron pool and increase Fenton-dependent oxidative stress; this does not consider that the fixation of iron accumulation as an entirely self-sufficient upstream trigger in a stationary cascaded manner. Whether it promotes ferroptosis requires that peroxidizable PUFA-PLs are available at the same time, and cellular antioxidant systems can inhibit lipid radical propagation. Identifying specific mechanisms of iron dyshomeostasis may define those subsets of tumors that are sensitive to ferroptosis, prioritize actionable regulatory nodes and rationalize mechanistically directed therapeutic development against ferroptosis.

### 3.2 Lipid peroxidation

Lipid peroxidation is the execution event of ferroptosis and is a signature mechanism that sets ferroptosis apart from other forms of programmed cell death, specifically apoptosis and autophagy. Ferroptosis is defined by iron-dependent, irreversible peroxidative damage to membrane phospholipids enriched in PUFAs, making it mechanistically distinct from other modes of cell death and ultimately leading to disruption of the plasma membrane. Of these, PUFA-PLs are the primary substrates and mediators of lipid peroxidation (Zhang J. et al., 2025; Park et al., 2025). Due to the presence of more than one double bond, PUFA-PLs are particularly sensitive to peroxidation at bis–allylic carbons and can be oxidised by labile iron or iron-containing enzymes to form relatively stable phospholipid hydroperoxides (PL-PUFA-OOHs) that transiently diffuse across membranes through oxidative chain processes (Qiu et al., 2024). In this context, the composition of lipids dictates the amount of oxidizable membrane substrates available, whereas peroxidative damage is modulated in parallel by labile iron availability and antioxidant detoxification capacity, including the GSH–GPX4 axis that reduces lipid peroxides such as PL-PUFA-OOH to their hydroxyl derivative PL-PUFA-OH. Ferroptotic membrane failure is a result of massive lipid peroxidation overload that exceeds lipid protection capacities, and not an isolated downstream event occurring subsequent to iron accumulation.

Lipid peroxidation is a dynamic and tightly regulated process, which relies on the concerted action of several lipid metabolic enzymes. Among them, ACSL4 has been revealed as a key modulator of the ferroptotic lipid metabolic network. ACSL4 catalyzes the conversion of PUFAs such as arachidonic acid (AA) and adrenic acid (AdA) to their respective acyl-CoA derivatives, allowing their incorporation into membrane phospholipids and increasing PUFA content of cellular membranes (Song et al., 2026). Also, higher ACSL4 expression or activity significantly increases lipid peroxide accumulation and ferroptotic cell death via amplification of membrane lipid oxidation, while inhibition of ACSL4 alters membrane composition, thus reducing lipid peroxidation status and suppressing ferroptosis (Tong J. et al., 2023). Thus, ACSL4 is not only a critical molecular driver of ferroptosis vulnerability but also a promising target for cancer therapy.

Lipid peroxidation is an essential effector of ferroptosis with intimate connections to tumorigenesis, tumor progression, and treatment response (Guo D. et al., 2026). Altered lipid metabolism can modify the phospholipid composition of membranes and the dynamics of lipid peroxidation to promote or inhibit ferroptosis in tumor cells involved in digestive system malignancies. As an example, in HCC, AA stimulates lipid peroxidation and induces ferroptosis via the SIRT5–ACSL4/LPCAT3/ALOX15 signaling axis (Xue et al., 2025). Furthermore, ACSL4 and cyclooxygenase-2 are generally upregulated during ferroptosis, together with increased intracellular lipid peroxidation; thus, their aberrant expression has been reported as common molecular markers of ferroptosis.

These interlinked processes collectively give rise to a lipid peroxidation phenotype that is essential for ferroptosis, including the accumulation of PUFA-PLs, membrane lipid remodeling mediated by ACSL4 and LPCAT3, phospholipid oxidation driven by ALOX15, and disruption of the GPX4-dependent antioxidant defense system. Consistent with this framework, reduced ACSL4 blocks the biosynthesis of PUFA-containing lipids, thereby inhibiting lipid peroxidation and ferroptosis (Zeng et al., 2023). Together, these processes establish a lipid-metabolic network that governs ferroptotic susceptibility and influences tumor progression and therapeutic response in digestive system cancers. Defining the mechanisms that govern lipid peroxidation may help identify therapeutically actionable nodes and inform the rational development of ferroptosis-targeted interventions in digestive system malignancies.

### 3.3 Antioxidant systems

Antioxidant systems are a central element of cellular redox defense and, hence, one of the most critical regulators of ferroptosis. Crucially, antioxidant capacity should not be considered a mere passive by-product of ferroptotic stress, as an elegant study has shown that the inhibition of GSH biosynthesis potentiated ferroptosis and sensitized cells to oxidative stress through ACTL6A ablation (Yang et al., 2023). Lipid peroxidation is an execution mechanism of ferroptosis, where lipid peroxides are locally generated in an iron-dependent manner until they break down membrane integrity and contribute to cell death. When GPX4-mediated antioxidant defense is impaired, lipid ROS and lipid peroxides build up, leading to membrane leakage and ferroptotic cell death (Zhou et al., 2024). Tumor cells are subjected to metabolically stressful and nutrient-poor microenvironments in digestive system tumors like PDAC, which become resilient by inducing ferroptosis-guarding factors such as SLC7A11 and GPX4, thus facilitating growth and expansion. Such adaptation can defend tumor cells from catastrophic lipid peroxidation under scarcity of the conditionally-essential amino acid, glutamine (Shen et al., 2025). Thus, the regulation of antioxidant systems not only correlates with ferroptosis sensitivity but might also provide therapeutic target vulnerabilities for exploiting ferroptosis in therapy that merit further preclinical and clinical studies.

Currently, the System Xc^−^–GSH–GPX4 axis represents one of the best-characterized antioxidant pathways involved in ferroptosis regulation. System Xc^−^, a cystine/glutamate exchange system consisting of the heterodimer formed from SLC7A11 and solute carrier family 3 member 2 (SLC3A2), mediates cystine transport across the plasma membrane in a one-to-one ratio with intracellular glutamate. Cystine imported is subsequently reduced to cysteine, which is the major substrate for GSH biosynthesis. GSH is one of the major non-enzymatic antioxidants in cells, and donates reducing equivalents for GPX4 that catalyzes lipid hydroperoxides into biologically neutral lipid alcohols, and not only terminates the propagation of lipid radicals but also protects membrane from peroxidation and maintains cellular homeostasis (Wu et al., 2026; Chen et al., 2026). Under glucose deprivation, impaired cysteine metabolism can reduce GSH availability and GPX4 activity, thereby promoting the accumulation of lethal lipid peroxides and ferroptotic cell death (Zhang M. et al., 2025). Conversely, in digestive system malignancies such as HCC, tumor cells exhibit a strong dependence on the GSH/GPX4 antioxidant machinery to eliminate lipid peroxides, evade ferroptosis, and maintain malignant progression (Chen Y. et al., 2025). Collectively, the System Xc^−^–GSH–GPX4 axis represents a core protective barrier against lipid peroxidation and a critical therapeutic vulnerability for ferroptosis induction in digestive system cancers.

In addition to GPX4-dependent antioxidant defenses, neoplastic cells may also resist ferroptosis via GPX4-independent pathways. One of these, the ferroptosis suppressor protein 1 (FSP1)/coenzyme Q10 (CoQ10) pathway has been shown to be a dominant antioxidant system integrated in parallel to GPX4. FSP1 is primarily membrane-bound and utilizes NAD(P)H to reduce oxidized CoQ10 to the lipophilic antioxidant ubiquinol. Ubiquinol is a very effective lipid radical scavenger, as well as an efficient chain-breaker of lipid peroxidation. Importantly, the FSP1/CoQ10 pathway is able to maintain membrane lipid homeostasis and inhibit ferroptosis even when GPX4 function is compromised (Wu et al., 2026; Chen Y. et al., 2025). Collectively, the GSH/GPX4 and FSP1/CoQ10 systems constitute a dual antioxidant network of lipid peroxidation protection within cells. The presence of this compensatory pathway also contributes to the innate resistance of some tumor cells to single-agent ferroptosis inducers. Additional GPX4-independent defense pathways, including the mitochondrial DHODH–CoQH2 and GCH1–BH4 systems, also contribute to ferroptosis resistance by limiting lipid peroxidation. These pathways indicate that ferroptosis suppression relies on multiple parallel antioxidant mechanisms rather than a single dominant pathway.

Besides the aforementioned antioxidant effectors, antioxidant activity is intricately regulated by multiple upstream signaling pathways and one of the well-studied transcriptional regulatory networks involving Kelch-like ECH-associated protein 1 (KEAP1)/NRF2 is a key regulator of the oxidative stress response, playing a crucial role in antioxidant transcriptional regulation by promoting cellular detoxification of ROS and lipid peroxides through transcriptional activation of diverse antioxidative genes, such as SLC7A11, GPX4, NQO1 and heme oxygenase-1 (HO-1), thereby inhibiting ferroptosis (Chen et al., 2026; Zhou et al., 2025). Aberrant NRF2 activation has been highly implicated in tumor aggressiveness, therapeutic resistance and poor prognoses in a wide range of the most common digestive system cancers. ZNF207 has also been reported to enhance tumor-cell resistance to oxidative stress and suppress ferroptosis by promoting antioxidant responses (Yang T. et al., 2025). Thus, ferroptosis resistance in tumor cells does not rely on isolated antioxidant molecules, but rather on the sustained and dynamic activation of coordinated antioxidant programs governed by transcriptional networks such as KEAP1/NRF2.

Antioxidant systems are central to preserving lipid redox homeostasis, particularly through the System Xc^−^–GSH–GPX4 axis, thereby inhibiting ferroptosis and promoting cell survival (Zhang et al., 2024). This is reflected by the remarkable reliance of tumors associated with digestive system on these antioxidant routes, which make them prospective therapeutic liabilities to augment anti-ferroptotic response. Characterization of the crosstalk and co-regulation between these pathways, coupled with assessment of multi-target combination strategies, may offer a biological rationale for designing precision-based translational approaches to treat digestive system cancers.

Overall, the central molecule of ferroptosis should not be seen as merely a linear chain where iron overload leads to lipid dysfunction and antioxidant collapse. Instead, ferroptotic susceptibility is due to the cooperation between disrupted iron homeostasis and impaired antioxidant defenses: expansion of the labile Fe^2+^ pool arising from defects in cellular iron storage and efflux causes lipid peroxidation, whereas NRF2 inactivation and associated repression of GCLC and SLC7A11 hamper GSH synthesis and GPX4-dependent redox defense thereby allowing deadly ROS-based accumulation by lipids (Zhu X. et al., 2023). These processes may occur simultaneously and together determine cell ferroptotic sensitivity: HMOX1-mediated labile iron accumulation promotes lipid peroxidation, while adaptive antioxidant responses—particularly GPX4-defended intracellular microenvironments that suppress overinduction of lipid peroxidation—influence whether oxidative insult evolves to ferroptosis (Shen et al., 2025). The reason for this is that ferroptosis arises when the integrated iron–lipid–redox network is driven beyond its buffering capacity, as opposed to activation of one linear cascade (Qiu et al., 2024). Together, these components create a mechanistic basis of the multilayered regulatory networks described below. In this way, it shows that ferroptosis-related factors cannot be treated as the same regulatory nodes. Ferroptotic cell death is dictated by core execution components and can be regulated by upstream factors that influence ferroptotic sensitivity. Changes in ROS, lipid peroxidation, or antioxidant markers should be viewed with caution and substantiated by functional rescue (e.g., ferroptosis inhibitors, antioxidants, or iron chelation can revert ferroptosis-related lipid peroxidation and cell death) (Zhou et al., 2024).

## 4 Multilevel regulatory network of ferroptosis

At the same time, these regulatory factors are not binary switches of ferroptotic cell death; rather, they work as modulators of ferroptotic thresholds dependent on tumor metabolic state, antioxidant capacity and cellular context. Collectively, sustained susceptibility to ferroptosis is reprogrammed by transcriptional, epigenetic, metabolic, post-transcriptional, immune, and TME-derived signals. Under the lactate-rich TME, H3K18la mediated by p300 promotes IGF2BP2 transcription to stabilize NRF2 mRNA and increase cell ferroptosis resistance in CRC cells (Zhu et al., 2025). Accordingly, this regulatory layer hence mediates the mechanistic link between core ferroptotic apparatus and cancer-type-specific resistance states curated in Section 5, which is illustrated in Figure 2.

### 4.1 Transcriptional and epigenetic regulation

Beyond alterations in iron homeostasis, ferroptosis in digestive system malignancies is governed by a complex, multilayered regulatory network. Transcriptional and epigenetic mechanisms—including RNA modifications, DNA methylation, histone modifications, chromatin remodeling and transcription-factor binding—reshape ferroptosis susceptibility by controlling the expression of key regulators such as SLC7A11, GPX4 and elongation of very long-chain fatty acids protein 5 (ELOVL5). These mechanisms interact with metabolic reprogramming and TME signals to coordinate redox homeostasis, lipid metabolism and therapeutic resistance. Crosstalk among these regulatory layers ultimately determines ferroptotic sensitivity and resistance.

Epitranscriptomic RNA modifications constitute a distinct regulatory layer of ferroptosis. Among these, N6-methyladenosine (m6A) modification influences mRNA stability, splicing, and translational efficiency, thereby modulating the expression of key ferroptosis-related genes. The m6A methyltransferase methyltransferase-like 3 (METTL3) mediates SLC7A11 transcription through GATA-binding protein 3 (GATA3) and lysine demethylase 6B (KDM6B), in an m6A-dependent manner, thus regulating susceptibility to ferroptosis (Zhang H. et al., 2025). SLC7A11 is a central component of System Xc^−^, which upregulates the uptake of cystine and subsequently directs GSH biosynthesis to maintain GPX4-mediated resistance to lipid peroxidation. Thus, the METTL3/SLC7A11 axis promotes antioxidant defense capacity and ferroptosis resistance in favor of tumor cells.

The hierarchical regulation of ferroptosis is additionally elaborated by histone modifications and chromatin remodeling. ELOVL5, which participates in fatty-acid elongation and PUFA metabolism, can increase the availability of peroxidizable lipid substrates when transcriptionally activated, thereby promoting ferroptosis. BAP1 is a nuclear deubiquitinase that is involved in epigenetic gene regulation by altering chromatin states (Fan et al., 2025). ATF7IP partners with SETDB1 to epigenetically silence transcription of CYB5R2 (Su et al., 2025). Histone methylation, lactylation, demethylation and chromatin silencing cooperate to govern the transcriptional landscape of ferroptosis-related genes, which offers an epigenetic basis for tumor cell heterogeneity in ferroptosis sensitivity.

Integration of transcriptional and epigenetic regulation connects cellular metabolic status, oxidative stress, lipid composition to cell-fate decisions through the regulation of gene expression related to both ferroptosis. Integration of molecular subtyping with epigenetic profiling may reveal novel ferroptosis-sensitive and ferroptosis-resistant tumor subgroups that inform the development of pathway-guided interventions and rational combination strategies.

### 4.2 The impact of metabolic reprogramming on ferroptosis

Metabolic reprogramming allows tumor cells and TME components to adapt through nutrient limitation and therapeutic pressure, it is also a key driver of ferroptosis sensitivity. The susceptibility to ferroptosis is tightly linked with the cellular metabolic state in a coordinated manner involving changes in iron metabolism, lipid peroxidation and antioxidant defense systems (CHEN W. et al., 2025). Changes in lipid metabolism, glycolysis and lactate accumulation, amino-acid metabolism, GSH biosynthesis and autophagy together orchestrate the sensitivity to ferroptosis in digestive system cancers.

Lipid metabolism reprogramming is one of the hallmarks underlying ferroptosis. Ferroptotic execution is a function of the gradual accumulation of lipid peroxides embedded in membrane phospholipids, with PUFA-PLs being the predominant target for peroxidation. ACSL4, a key enzyme in the synthesis of PUFA-PL, is related to lipid metabolism and acts as an important molecular determinant of sensitivity to ferroptosis (Zeng et al., 2023). PUFA-mediated ferroptosis inhibits pancreatic cancer cell proliferation, and increasing the pool of oxidizable membrane lipids may make cells more susceptible to ferroptosis (Suda et al., 2024). Besides substrate abundance, lipid-associated co- and post-translational modifications can also stabilize key ferroptosis-related proteins. For example, palmitoylation is important in stabilizing GPX4 and alters the cell sensitivity to ferroptosis (Huang B. et al., 2025).

Regulation of ferroptosis is also mediated by lactate production through enhanced glycolysis. Lactate is not only the dominant end-product of glycolytic metabolism in cancer, but also acts as a signaling metabolite that regulates gene expression by way of histone lactylation. In addition, lactate-mediated lactylation has been shown to inhibit ferroptosis in CRC stem cells (Deng J. et al., 2025), and high glycolytic activity may provide tumor cells with an epigenetic mechanism that promotes resistance to ferroptosis. In turn, histone deacetylase (HDAC) inhibitors increase the ferroptotic sensitivity of CRC cells, at least in part by reducing K412 lactylation of HDAC1 (Yang et al., 2025c). Taken together, the lactate–lactylation axis represents a novel integrative role between metabolic reprogramming, tumor stemness maintenance and ferroptosis resistance.

Ferroptosis susceptibility is also modulated and shaped by rewiring of amino acid metabolism and antioxidant systems. Cystine is a limiting substrate for GSH biosynthesis while GSH is an indispensable cofactor for GPX4 activity modulation and lipid peroxide detoxification. Through DUB-IN-3 treatment or ubiquitin-specific protease 8 (USP8) depletion, intracellular cystine availability and GSH biosynthesis will be inhibited, promoting accumulation of ROS, inhibiting antioxidant capacity in cells and increasing sensitivity to ferroptosis (Tang et al., 2023a). In addition, oncogenic and metabolic signaling pathways are also able to reverse the sensitivity of ferroptosis. For instance, S100P modulates lipid metabolic pathways and inhibits ferroptosis in HCC (Yang M. et al., 2025). In another HCC study, prevention of PGAM1 catalytic function led to ferroptosis via energy stress and AKT inhibition that depended on ROS production resulting in LCN2 downregulation; this also increased CD8^+^ T-cell infiltration and augmented anti-PD-1 effectiveness in preclinical models (Zheng Y. et al., 2023).

Collectively, metabolic reprogramming shapes ferroptosis susceptibility by regulating lipid substrates, lactate/lactylation, cysteine–GSH metabolism, and ROS homeostasis. These changes across fatty-acid, glycolytic, amino-acid, and oncogenic metabolic pathways may define ferroptosis-responsive tumor subsets and inform combination strategies. Hypoxia further modulates ferroptosis in a context-dependent manner: acute hypoxia may increase oxidative vulnerability, whereas chronic hypoxia can promote resistance through metabolic adaptation, NRF2 activation, and altered lipid metabolism, with effects varying by tumor type, nutrient availability, exposure duration, and cellular compartment.

### 4.3 Regulatory roles of the TME in ferroptosis

The TME consists of tumor cells, immune cells, fibroblasts, and extracellular components and various metabolites and signaling factors that modulate tumor advancement, ferroptosis vulnerability/insensitivity as well as therapeutic resistance. In addition to being modulated by various intracellular metabolic pathways, ferroptosis activity is regulated by the immune status of TME and intercellular communication with TME (Wang et al., 2019). Microenvironmental signals tightly control lipid peroxidation, oxidative stress, and antioxidant defenses to calibrate tumor-cell ferroptosis sensitivity, disease progression, and therapeutic response.

Immune cells serve as a critical modulatory element of ferroptosis in the TME. CD8^+^ T cell–mediated antitumor immunity, in particular, is closely connected to ferroptosis. Activated CD8^+^ T cells are known to release interferon-γ, which inhibits the expression of SLC7A11, a core component of System Xc^−^ that restricts cystine uptake and GSH synthesis. This cascade facilitates lipid peroxide expansion and eventually leads to ferroptosis of tumor cells (Wang et al., 2019). This mechanism provides an important link between antitumor T-cell activity and enhanced ferroptotic susceptibility of tumor cells. Moreover, groups of immunosuppressive cells also participate in regulating iron-dependent cell death by modulating TME inflammatory state through cytokine release and oxidative stress levels that define tumor cell fate in GC (Sert and Ulucan, 2025).

In addition to immune regulation, intercellular communication mediated by exosomes represents another important way that TME regulates ferroptosis. They contain proteins, lipids and nucleic acids that allow bidirectional trafficking between tumor cells and stromal elements. Exosome-derived SLC1A5 is known to inhibit GPX4-dependent ferroptosis, supporting GC progression (Zhang et al., 2025d). These findings suggest that tumor cells may exploit microenvironment-derived metabolic and antioxidant support to maintain ferroptosis resistance. Conversely, ferroptotic tumor cells can, in specific contexts, release signals that contribute to a tumor-protective microenvironment (Mbah et al., 2026). Ferroptosis-associated signals, including various lipid-derived metabolites and inflammatory cues, may also create an immunosuppressive environment that allows tumors to grow while evading therapeutic efficacy.

Collectively, ferroptosis and the TME engage in reciprocal regulation: microenvironmental signals shape ferroptotic susceptibility, whereas ferroptotic tumor cells can release damage-associated and lipid-derived signals that alter immune and stromal responses (Dai et al., 2020). Consequently, ferroptosis opens an important window into the overlapping biology of tumor metabolism, immune regulation, and microenvironmental remodeling; understanding these reciprocal interactions could inform therapeutic strategies targeting both ferroptosis susceptibility and TME. Of central importance, the biological significance of ferroptosis is highly linked with the cellular compartment that is influenced. Ferroptosis in malignant epithelial cells is usually related to the removal of tumor-cell, but ferroptotic injury to antitumor immune cells or normal parenchymal cells could more likely subvert immuno-surveillance or damage tissue tolerance (Kim et al., 2022). The stromal and endothelial compartments may additionally modulate tumor-cell ferroptosis based on nutrient availability, redox buffering, lipid exchange, inflammatory signaling and vascular stress. Therefore, the therapeutic success of ferroptosis is not solely related to whether or not ferroptosis occurs, but also in what cellular compartment ferroptotic stress occurs and how bystander cells suffice.

### 4.4 Interplay among distinct regulatory mechanisms

Ferroptosis is governed by a multilayered network of oxidative stress, iron and lipid metabolism, autophagy, post-translational modifications, and tumor immunity. These interconnected processes collectively determine ferroptosis susceptibility and contribute to tumor progression, therapeutic resistance, and TME remodeling in digestive system malignancies, highlighting their importance as potential targets for combinatorial therapeutic strategies.

Redox homeostasis serves as a major point of convergence among multiple ferroptosis-regulatory pathways. ROS play a direct role in lipid peroxidation, but also indirectly regulate the sensitivity of cells to ferroptosis by post-translational modifications and transcriptional networks (Wang S. et al., 2025). For example, CST1 recruits the deubiquitinase OTU deubiquitinase, ubiquitin aldehyde binding 1 (OTUB1), thereby reducing GPX4 ubiquitination and stabilizing GPX4. This enhances lipid-peroxide detoxification, suppresses ferroptosis, and contributes to GC metastatic progression. Furthermore, O-GlcNAc transferase (OGT)-mediated O-GlcNAcylation of SLC7A11 regulates cystine uptake and GSH synthesis, thereby enhancing antioxidant capacity and promoting ferroptosis resistance in tumor cells (Tang et al., 2023a). These post-translational modifications are closely integrated with redox regulation and collectively modulate GPX4 stability, SLC7A11-mediated cystine uptake, and ROS homeostasis, thereby shaping ferroptosis susceptibility.

Autophagy, antioxidant signalling and mitochondrial homeostasis are also closely linked. In HCC, mitochondrial TSPO interacts with P62 to interrupt the autophagic flow and gives rise to accumulation of P62. Accumulated P62 competes with NRF2 for KEAP1 binding and reduces KEAP1-dependent proteasomal degradation of NRF2 (Zhang et al., 2023). As a consequence, stabilized NRF2 upregulated a number of antioxidant genes, which facilitate cell adaptation to oxidative damage and also protect cells against ferroptosis. In addition, cGAS deficiency or DRP1 oligomerization inhibition promotes mitochondrial ROS accumulation and ferroptosis to inhibit tumor growth (Qiu et al., 2023). Such findings uncover a selectively regulated feedback loop connecting autophagy dysregulation, impaired mitochondrial dynamics and oxidative stress to govern the fate of tumor cells.

Ferroptosis is also regulated by the interaction of iron metabolism and tumor immunity. CCT3 interacts with ACTN4 and inhibits the recycling of transferrin receptor 1 (TFRC) to the plasma membrane, ultimately leading to reduced iron uptake and decreased sensitivity to ferroptosis (Zhu et al., 2024). Combining USP8 inhibition with ferroptosis inducers suppresses tumor growth and promotes CD8^+^ T-cell infiltration into the TME (Li H. et al., 2024), suggesting that disruption of ferroptosis-resistance mechanisms may enhance antitumor immunity. In a similar fashion, combination treatment with donafenib and the histone demethylase inhibitor GSK-J4 led to the synergistic upregulation of heme oxygenase 1 (HMOX1) expression and enhancement of intracellular Fe^2+^ levels, thus leading to ferroptosis (Zheng C. et al., 2023). These observations underscore that multi-pathway interventions can enhance ferroptotic responses through coordinated regulation of iron metabolism, oxidative stress, and immune activity.

Together, the ferroptotic response is supported by a multidimensional network of signaling regulation that includes oxidative stress, post-translational modifications, autophagy, mitochondrial homeostasis, iron metabolism and immune modulation. These mechanisms can interact in a cooperative or antagonistic manner leading to cellular responses to ferroptotic stimuli. More mechanistic studies could corroborate the preclinical testing of multi-axis-targeting approaches and certification of candidate regulatory nodes for future biomarker-guided therapeutic options in gastrointestinal malignancies.

## 5 Roles and context-dependent effects of ferroptosis across digestive system cancers

The second layer of framework is cancer-type specific ferroptosis resistance. Despite the converging iron–lipid–redox execution machinery in CRC, HCC, GC and PDAC, they activate unique resistance programs based on various aspects of iron metabolism, lipid availability, redox regulation by antioxidant defenses, oncogenic signaling and microenvironmental reprogramming. Comparison of these mechanisms highlights common weaknesses, tumor-specific escape routes and candidate therapeutic susceptibility markers summarized in Figure 3 and Table 3.

**FIGURE 3:**
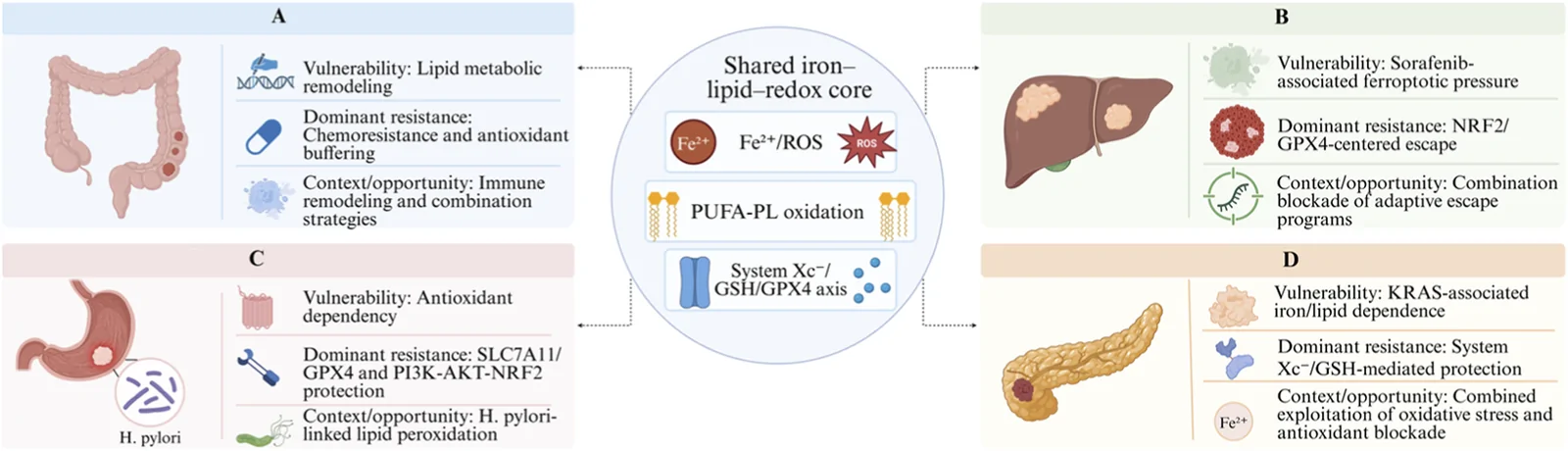
Comparative framework of ferroptosis regulation across core digestive system malignancies. This Review points out shared mechanistic principles of ferroptotic regulation across CRC, HCC, GC and PDAC through iron availability, oxidation of PUFA-containing membrane phospholipids and antioxidant defense systems (especially the System Xc^−^–GSH–GPX4 axis) while uncovering interesting cancer-type-specific vulnerabilities as well. Ferroptosis sensitivity and its roles as a foundation for chemotherapy resistance in CRC can be attributed to the reprogramming of lipid metabolism. NRF2–GPX4 signaling, RNA processing and post-translational regulation contribute to ferroptosis susceptibility and sorafenib response in HCC. SLC7A11–GPX4 and PI3K/AKT–NRF2 signaling reinforce antioxidant defense against *helicobacter* pylori-induced lipid peroxides regulating sensitivity to ferroptosis in GC. Metabolic regulation mediated by KRAS, System Xc^−^-GSH-mediated protection and iron–lipid metabolic dependencies synergistically dictate ferroptosis sensitivity in PDAC. Collectively, these overlapping and biallelic mechanisms are involved in tumor evolution, improvement of therapy resistance,3 and sensitivity to ferroptosis-modulating strategies. Abbreviations: CRC, colorectal cancer; GC, gastric cancer; GPX4, glutathione peroxidase 4; GSH, glutathione; HCC, hepatocellular carcinoma; KRAS, Kirsten rat sarcoma viral oncogene homolog; NRF2, nuclear factor erythroid 2-related factor 2; PDAC, pancreatic ductal adenocarcinoma; ROS, reactive oxygen species; SLC7A11, solute carrier family 7 member 11; System Xc^−^: cystine/glutamate antiporter system. The comparative features of these malignancies, including molecular drivers, metabolic dependencies, therapeutic context, and evidence strength, are highlighted to distinguish established mechanisms from emerging vulnerabilities. **(A)** CRC (Colorectal cancer). **(B)** C, GC (Gastric cancer). **(C)** HCC (Hepatocellular carcinoma). **(D)** PDAC (Pancreatic ductal adenocarcinoma).

**TABLE 3:** Comparative ferroptosis landscape across the four core digestive system malignancies.

Cancer	Driver genotype	Iron dependence	Lipid state	Antioxidant dependency	TME	Treatment context	Evidence level	Consequence
CRC	APC/KRAS/BRAF	TFRC/iron metabolism	ACSL4/PUFA	SLC7A11-GPX4	immune/macrophage	chemotherapy resistance	preclinical	mainly antitumor
HCC	TP53/β-catenin	iron metabolic vulnerability	ACSL4/LPCAT3	NRF2-GPX4	immune inflammatory	sorafenib	preclinical/human association	therapeutic vulnerability
GC	PI3K/AKT/HER2	iron uptake	lipid oxidation	SLC7A11-GPX4	H.pylori inflammation	chemotherapy	preclinical	mainly antitumor
PDAC	KRAS	TFRC-dependent iron	PUFA metabolism	System Xc-GSH	stroma-rich TME	chemotherapy	preclinical	context dependent

This table is an author-generated qualitative synthesis based on the evidence discussed in Sections 5.1–5.4. Representative references already cited in the corresponding sections include: CRC (Li et al., 2025; Wang S. et al., 2025; Yang Q. et al., 2025; Xin et al., 2024); HCC (Zhang B. et al., 2022; Li et al., 2023; Liu et al., 2024; Li Q. et al., 2024); GC (Jiang et al., 2023; Huang et al., 2024; Zhu W. et al., 2023); and PDAC (Zhao H. et al., 2025; Xu et al., 2024; Ma et al., 2023; Suda et al., 2024).

### 5.1 Functional characteristics of ferroptosis in CRC

CRC is one of the most prevalent gastrointestinal malignant diseases closely linked to metabolic reprogramming, redox imbalance and/or altered microenvironmental signaling. Ferroptosis is involved in CRC progression and treatment response based on several tumor-intrinsic and microenvironmental mechanisms. As such, inhibin beta A can contribute to CRC tumorigenesis by promoting macrophage M2 polarization and suppressing mitochondria-dependent ferroptosis (Li et al., 2025). Because of metabolic plasticity and dynamic antioxidant programs in CRC cells, ferroptosis susceptibility is modulated by cellular context and treatment conditions. Elucidating these regulatory features may help identify therapeutic vulnerabilities and inform combinatorial treatment strategies.

Ferroptosis is directly linked to the proliferation and migratory function of CRC cells. Decreased ILF3 inhibits CRC cell proliferation and at the same time increases ferroptosis sensitivity (Wang S. et al., 2025). Similarly, villin 1 knockdown induces ferroptotic cell death and reduces CRC cell migration (Hu et al., 2025), suggesting that ferroptosis is not only involved in regulating tumor cell survival but also tightly intertwined with invasive and metastatic potential. Fatty acid desaturase 2 (FADS2) has been implicated in the transcriptional regulation and activity of several lipid metabolism–associated enzymes, and it was demonstrated that knockdown of FADS2 altered ferroptotic processes in CRC cells (Yang Q. et al., 2025). Given the central role of lipid peroxidation in ferroptosis execution, lipid metabolic reprogramming likely represents a critical determinant of ferroptotic vulnerability in CRC.

Adaptation to ferroptosis in CRC is further implicated in the development of chemoresistance. LncRNA-HMG has been reported to protect CRC cells from chemotherapy-induced ferroptosis, thereby promoting drug resistance (Xin et al., 2024). In addition, heterogeneous nuclear ribonucleoprotein C reduces sensitivity to irinotecan (CPT-11) by suppressing ferroptosis in CRC cells (Zhu et al., 2026). Evasion of ferroptosis may therefore constitute an important mechanism underlying chemotherapy resistance in CRC, and targeting ferroptosis-regulatory nodes may enhance chemosensitivity in experimental models and warrant further translational evaluation.

Beyond endogenous regulatory circuits, a range of bioactive natural compounds and emerging therapeutic strategies can induce ferroptosis to exert anti-CRC effects. Ursolic acid has been shown to trigger ferroptosis in CRC cells by downregulating key components of System Xc^−^, including SLC7A11, SLC3A2, and GPX4, thereby markedly inhibiting tumor proliferation (Shi et al., 2025). Likewise, delavinone induces ferroptosis through inhibition of protein kinase Cδ and suppression of NRF2 phosphorylation, contributing to its anti-CRC activity (Zhang et al., 2025e). In recent years, ferroptosis-based combination strategies have gained increasing attention in CRC therapy. A dual-targeted GOx@FeNPs nanoplatform enables synergistic photothermal therapy and ferroptosis induction, thereby further potentiating antitumor immune responses (Li et al., 2024b). This strategy enhances tumor susceptibility to ferroptosis and remodels the tumor immune microenvironment in preclinical models, supporting further translational evaluation.

Data to date provides support for CRC ferroptotic vulnerability primarily derived from lipid-peroxide regulation and antioxidant buffering capacity, while resistance under therapeutic pressure may reflect a combination of metabolic plasticity and activation of ferroptosis-suppressive programs. Therefore, it shall be indicated to test the combinatorial strategy of ferroptosis induction with chemotherapy and selected nano- or immune-modulatory approaches in suitable preclinical ferroptosis-responsive CRC models as well as candidate molecular subgroups.

### 5.2 Characteristics of ferroptosis in HCC

HCC is a major malignancy of the digestive system, and chronic inflammation, metabolic dysregulation, and oxidative stress are important contributors to its development and progression. Since the liver is a central organ for systemic iron and lipid metabolism, HCC cells are highly susceptible to ferroptosis triggered by dysregulated intracellular iron homeostasis (Zhang B. et al., 2022). Evidence is rapidly emerging linking ferroptosis to HCC cell proliferation, tumor progression and therapeutic resistance, with sorafenib-induced ferroptotic death being a major focus in the field of HCC research (Louander et al., 2013). Defining the regulatory mechanisms of ferroptosis in HCC may help clarify determinants of therapeutic response and resistance and inform more individualized treatment strategies.

Ferroptosis induced by sorafenib is a clinically-relevant mechanism of action in HCC and serves as a basis to comprehend both cancer-type specificity, and resistance mechanisms for ferroptosis. Although sorafenib activates RFX1-mediated ferroptotic signaling and induces death of HCC cells (Yang et al., 2025f), the long-term therapeutic pressure drives diverse adaptive programs in HCC cells that converge on inhibition of lipid peroxidation and ferroptosis execution, therefore limiting sorafenib efficacy. A predominant route of this network of resistance is increased antioxidant response and reduction of lipid-peroxide levels. Sorafenib promotes NRF2 activation, what inhibits both lipid peroxidation and ferroptosis leading to drug resistance (Liu et al., 2024). DDX39B also facilitates GPX4 pre-mRNA splicing and cytoplasmic export, resulting in higher levels of GPX4 expression and improved detoxification of lipid-peroxides which protects HCC cells from sorafenib-induced ferroptosis (Li Q. et al., 2024). Restoration of the antioxidant buffering through NRF2- and GPX4-associated mechanisms, is one major way in which HCC cells escape the sorafenib-induced ferroptotic pressure as revealed by these findings.

Further resistance pathways are mediated by oncogenic signaling and metabolic control. Sorafenib promotes DDX5 downregulation which induces Wnt/β-catenin signaling and shifts HCC cells toward ferroptosis resistance, decreasing sorafenib sensitivity (Li et al., 2023). Loss of SLC27A5 also reduces sorafenib induced ferroptosis and confers drug resistance (Xu F. L. et al., 2023). So, antioxidant reinforcement, RNA-processing-dependent GPX4 regulation, Wnt/beta-catenin signalling, and disturbance of lipid metabolic control should be viewed as related escape modules converging on decreased ferroptotic execution rather than as unrelated molecular events.

In addition to these mechanisms immediately associated with sorafenib, certain epigenetic and post-translational programs can also adjust the ferroptotic threshold in HCC which may ultimately impact the broader resistance state. m6A-dependent RNA regulation is related to the ferroptosis in HCC (Li et al., 2024d), and USP8-mediated deubiquitination promotes β-catenin stabilization by making cells resistant to ferroptosis (Tang et al., 2023b). These regulatory layers may cooperate with antioxidant, RNA-processing, oncogenic and metabolic programs to define a cell’s state nonpermissive to ferroptotic execution.

This network has therapeutic implications in pushing back sorafenib-resistant disease by simultaneously restoring ferroptotic pressure while interrupting adaptive survival programs. For instance, metformin induces ferroptosis through the ATF4/STAT3 axis, promotes intracellular ROS and lipid peroxidation, and increases HCC-cell sensitivity to sorafenib (Hu et al., 2023). These findings support combination strategies that target ferroptosis-escape mechanisms rather than relying on sorafenib-induced ferroptosis alone.

Recent evidence suggests that the response to sorafenib in HCC is determined by the interplay between signals promoting ferroptosis and those counteracting it with adaptive resistance mechanisms. While sorafenib can induce ferroptotic cell death, NRF2-dependent antioxidant activation, DDX39B-mediated maintenance of GPX4, DDX5–Wnt/β-catenin signaling, loss of SLC27A5 and YAP/TAZ–ATF4 signaling all converge through distinct regulatory pathways to block lipid peroxidation or execution of ferroptosis, thus lowering sensitivity to sorafenib (Gao et al., 2021). This ferroptotic threshold is further modified by epigenetic, post-translational and metabolic regulators, providing a rationale for the combination of sorafenib with interventions to disable dominant ferroptosis-escape mechanisms in biologically defined HCC contexts. Conclusively, ferroptosis in the context of sorafenib therapy should not be recognized as a causative driver of sorafenib response but rather as context-dependent, based on dosage, time of exposure, genetic background, and adaptive resistance mechanisms.

### 5.3 Functional features of ferroptosis in GC

GC is a common human malignancy of the digestive system, and its initiation and progression are closely related to chronic inflammation, redox imbalance and abnormal lipid metabolism. Ferroptosis, an iron-dependent and lipid peroxidation-driven regulated cell death pathway, plays critical roles in GC progression, particularly in tumor growth and chemoresistance (Zhang et al., 2020). Because GC cells exhibit heterogeneous antioxidant capacities, their susceptibility to ferroptosis varies substantially. Defining the underlying regulatory mechanisms may therefore facilitate the development of more effective therapeutic strategies.

Ferroptosis in GC is closely associated with disrupted antioxidant defense systems. GSH metabolism helps maintain intracellular redox homeostasis in GC cells, thereby limiting lipid peroxidation and promoting ferroptosis resistance. ACTL6A–induced expression of glutamate-cysteine ligase catalytic subunit decreases ROS levels, ameliorates lipid peroxidation and suppresses ferroptosis (Yang et al., 2023). Meanwhile, SLC7A11 as a major component of System Xc^−^ inhibits ferroptosis through activation of the PI3K/AKT signaling pathway, which contributes to the survival of GC cells (Jiang et al., 2023). In addition, transcription factor activating transcription factor 2 (ATF2) can initiate heat shock protein HSPH1 expression and stabilize SLC7A11 protein to augment GSH biosynthesis, inhibit ferroptosis and ultimately diminish GC sorafenib sensitivity. This raises the possibility that circumventing ferroptosis may be a major underlying mechanism of therapeutic resistance in GC (Xu X. et al., 2023). Aberrant activation of antioxidant defense networks therefore confers a substantial survival advantage and promotes tumor progression and therapeutic resistance.

Ferroptosis is also closely associated with chemoresistance in GC. Yi-Qi-Hua-Yu-Jie-Du Formula has been shown to induce ferroptosis in cisplatin-resistant GC cells by suppressing GPX4 expression via the AKT/GSK3β/NRF2 pathway, thereby attenuating chemoresistance (Huang et al., 2024). Similarly, red ginseng polysaccharides promote ferroptosis through inhibition of the PI3K/AKT signaling pathway (Wang et al., 2024). These findings indicate that PI3K/AKT and NRF2-mediated antioxidant signaling contributes substantially to ferroptosis resistance and chemoresistance in GC, potentiating their inhibition as therapeutic targets for counteracting drug resistance. Natural bioactive compounds have also shown ferroptosis-promoting activity in experimental GC models. Quercetin and tanshinone I have been reported to promote ferroptosis and suppress GC-cell growth (Ding et al., 2024). Other studies have implicated KDM4D suppression and p53 signaling activation, accompanied by downregulation of SLC7A11 and GPX4, in ferroptosis induction in GC (Xia et al., 2023). Amentoflavone can likewise suppress proliferation and promote ferroptotic cell death through the miR-496/ATF2 axis (Tang F. et al., 2023). These findings support natural products and their derivatives as candidate ferroptosis modulators for further preclinical evaluation in GC.

Under intrinsic antioxidant regulation and drug resistance mechanisms, ferroptosis in GC is also characterized by disease-specific features, especially in the context of *Helicobacter pylori* infection. *H. pylori* infection increases ferroptosis sensitivity in GC cells by upregulating PHKG2 expression and enhancing ALOX5-mediated lipid peroxidation (Zhu W. et al., 2023). Infection-induced inflammation promotes gastric carcinogenesis and regulates ferroptosis via regulating the iron-mediated lipid metabolism and oxidative stress responses. The pro-inflammatory milieu elicited by *H. pylori* in turn modifies cellular redox equilibrium and lipid metabolism to modulate sensitivity to ferroptosis. The molecular mechanisms underlying the interplay among infection, inflammation, and ferroptosis remain incompletely understood and warrant further investigation.

In summary, a central ferroptotic vulnerability of GC is that active dependence on SLC7A11–GSH–GPX4 and PI3K/AKT–NRF2-mediated antioxidant protection is the defining feature of these cells, whilst enhancement of these redox-defense pathways constitutes a master switch regulating both ferroptosis resistance and chemoresistance. Thus, concurrent blockade of anti-oxidative defenses in combination with standard chemotherapies or other features inducing ferroptosis may provide a new and promising approach for sensitivity to ferroptotic therapy in specific contexts of GC oncogenesis (Zhang et al., 2020; Jiang et al., 2023; Huang et al., 2024).

### 5.4 Functional features of ferroptosis in pancreatic cancer

Late diagnosis and pronounced chemoresistance make PDAC one of the most aggressive malignancies among digestive neoplasms. Because of its close interaction with tumor growth, invasion, metastasis, KRAS-driven metabolic reprogramming, antioxidant adaptation and anticancer therapy resistance, ferroptosis has received growing interest in PDAC. Due to the close association of pancreatic cancer cells with redox homeostasis and iron metabolism, disclosing ferroptosis-regulated networks can expose a dependency or vulnerability, or facilitate therapeutic strategies.

KRAS-dependent reprogramming of metabolism generates a context-specific state amenable to ferroptosis in PDAC, rather than constitutive promotion or inhibition of ferroptosis. On the one hand, KRAS/FOSL1/TFRC axis connects oncogenic KRAS signaling to dysregulated iron metabolism and promotes sensitivity to ferroptosis by TFRC-dependent iron addiction (Zhao H. et al., 2025). Conversely, KRAS-mutant PDAC cells may develop compensatory resistance pathways that limit lipid peroxidation while maintaining redox balance. MGST1 upregulation, for instance, inhibits lipid peroxidation-dependent ferroptosis and protects KRASG12D-mutant pancreatic cancer cells from MRTX1133-mediated cytotoxicity (Xu et al., 2024). For this reason, it should not be concluded from the apparently conflicting regulation of ferroptosis by KRAS that KRAS causes ferroptosis through antagonistic intrinsic effects. Oncogenic KRAS thus drives tumor-promoting metabolic reprogramming while at the same time imposes iron- and lipid-dependent metabolic liabilities that might increase susceptibility to ferroptosis; whether this vulnerability ultimately leads to ferroptotic cell death depends on the activation of compensatory antioxidant and lipid protective mechanisms.

In the context of the metabolic landscape associated with KRAS, antioxidant buffering represents a critical counterpoint to lipid- and iron-mediated ferroptotic stress. Knockdown of SLC3A2 or inhibition of 4F2hc N-glycosylation inhibits System Xc^−^ activity and reduces intracellular GSH levels, increasing the sensitivity of PDAC cells to ferroptosis (Ma et al., 2023). These findings indicate that PDAC cells depend on System Xc^−^–GSH-mediated redox protection to counterbalance the ferroptotic liability imposed by increased iron dependence and peroxidizable lipid stress, establishing antioxidant defense as a co-determinant of ferroptosis susceptibility rather than an isolated pathway. Overall, KRAS-mediated metabolic reprogramming integrates the components of iron–lipid–redox axis by influencing how PDAC manages oxidative stress via TFRC, lipid peroxidation, and System Xc- GSH to exploit or evade ferroptosis. This KRAS-ferroptosis connection is further modulated by additional signaling pathways. NOTCH2-dependent ferroptosis inhibition contributes to the progression of pancreatic cancer (Zhang S. et al., 2025), while SOX10 knockdown enhances sensitivity to ferroptosis and inhibits tumor growth through CMTM7-mediated Wnt/β-catenin signaling pathway (Jiang G. et al., 2025). Accordingly, these pathways should be interpreted as co-modulators of the ferroptosis-resistant state instead of independent core mechanisms. Overall, this stringent dependence on antioxidant systems highlights the emerging centrality of the System Xc^−^–GSH–GPX4 axis in the context of PDAC as a unique point of vulnerability.

This integrated vulnerability provides a mechanistic rationale for combination approaches that simultaneously increase iron-dependent oxidative pressure and weaken antioxidant protection. For example, Fe-doped hydroxyapatite combined with the GPX4 inhibitor RSL3 strongly induces ferroptosis in KRAS-mutant PANC-1 cells (Lomphithak et al., 2024), illustrating how iron loading and disruption of GPX4-dependent defense can cooperatively exploit the ferroptotic liability of KRAS-mutant PDAC. PUFAs induce lipid peroxidation and elicit antitumor effects in both gemcitabine (GEM)-sensitive and GEM-resistant pancreatic cancer (Suda et al., 2024). Besides, the natural compound erianin triggers cell cycle arrest and ferroptosis in pancreatic cancer cells with substantial inhibition of their viability (Qu et al., 2025). Together, these studies indicate that combination strategies enhancing lipid peroxidation vulnerability, iron metabolism, and antioxidant defense networks may improve therapeutic responses and help overcome chemoresistance in pancreatic cancer.

Thus, KRAS-mutant PDAC displays a context-dependent ferroptotic vulnerability, borne from evasion of oncogenic metabolic reprogramming, rather than a generalized pro- or anti-ferroptotic genotype. KRAS-related changes in iron handling and lipid metabolism lead to higher levels of iron-driven oxidative stressors as well as peroxidizable lipid substrates, while adaptive central pathways such as MGST1-mediated suppression of lipid peroxidation and the System Xc^−^–GSH–GPX4 antioxidant system shield this susceptibility by contributing to ferroptosis resistance. Therefore, therapeutic strategies that simultaneously engage with iron/lipid metabolic dependence while disabling compensatory antioxidant defenses are deserving of further clinical evaluation to overcome treatment resistance in molecularly-defined KRAS-mutant PDAC contexts.

### 5.5 Emerging evidence beyond the four core digestive system malignancies

While this Review focuses on four archetypal cancers (CRC, HCC, GC and PDAC) in a cancer-type specific manner, growing evidence from other digestive tract tumors also supports the notion of broad applicability and intrinsic variability to ferroptosis regulation. Since the available evidence regarding ferroptosis in these additional cancers is still more limited, they are classified under emerging evidence rather than as independent cancer-specific sections. In ESCC, ZIP8 is upregulated and functions to increase intracellular Zn^2+^ levels and promote CREB phosphorylation, which would enhance GPX4 expression and inhibit lipid peroxidation and ferroptosis (Yang et al., 2025g).

In contrast, knockdown or pharmacological inhibition of ZIP8 reduces GPX4 expression and increases lipid peroxidation and iron accumulation/ferroptosis. In the extrahepatic cholangiocarcinoma, the AKR1C1–CYP1B1–cAMP–PKA signaling axis has been proposed to regulate ferroptosis (Liu C. et al., 2025). AKR1C1 overexpression decreases the accumulation of lipid ROS and Fe^2+^, thus rendering cells resistant to ferroptosis; in contrast, knockdown of AKR1C1 increases cellular lipid peroxidation and sensitizes cells to ferroptosis inducers erastin and RSL3. These data demonstrate generality in GPX4-centric antioxidant defence and lipid peroxidation as evolutionarily conserved ferroptotic determinants across digestive system malignancies with tumour-specific regulatory impact. Because of limited evidence in ESCC, extrahepatic cholangiocarcinoma and other digestive tumors receiving less attention, these studies are described as emerging evidence that supplement but do not replicate the four primary cancer types.

## 6 Therapeutic strategies targeting ferroptosis and recent advances

The third layer of the framework relates to the process of therapeutic translation. Instead of evaluating ferroptosis-targeted interventions as a singular class, the following strategies are stratified by vulnerability target, the predominant resistance mechanism they overcome, and candidate biomarkers for potential patient selection. This mechanistic concordance is critical because the sensitivity of cells or malignancies to ferroptosis is context-dependent, and most current therapeutic interventions are still preclinical or investigational (Doll et al., 2017).

The therapeutic translation of ferroptosis-based strategies is strongly cancer-type dependent. The existing evidence is quite mature in the field of HCC, especially for sorafenib-associated ferroptosis (Louander et al., 2013), while the evidence from CRC, GC and PDAC is mainly preclinical. Different tumor types display heterogeneity in terms of unique ferroptotic vulnerabilities: lipid metabolic remodeling (CRC), dependence on antioxidants (GC), and KRAS-associated metabolic adaptation to ferroptosis stress (PDAC). Thus, ferroptosis-targeted therapeutic strategies should take into account tumor-specific molecular contexts rather than being globally applied to digestive system malignancies.

### 6.1 Ferroptosis inducers

Potential strategies for inducing ferroptosis constitute a new and exciting treatment concept in the field of digestive system malignancies; these strategies possess varying degrees of translational readiness. They may be broadly divided into experimental ferroptosis inducers, clinically approved drugs with potential as indirect modulators of ferroptosis-associated pathways, and therapies that have been clearly validated in the clinic through specific mechanisms of ferroptosis (Zhang et al., 2025g). Unlike apoptosis-based therapies, ferroptosis induction may provide an alternative strategy to bypass apoptosis resistance and overcome therapy-resistant tumor states, highlighting its translational potential in GC, HCC, and pancreatic cancer (Hangauer et al., 2017). Ferroptosis-targeted therapy includes pharmacological induction, nanomaterial-based delivery and combination regimens. Its clinical translation, however, remains limited by tumor heterogeneity, context-dependent resistance and the lack of validated biomarkers, as summarized in Figure 4.

**FIGURE 4:**
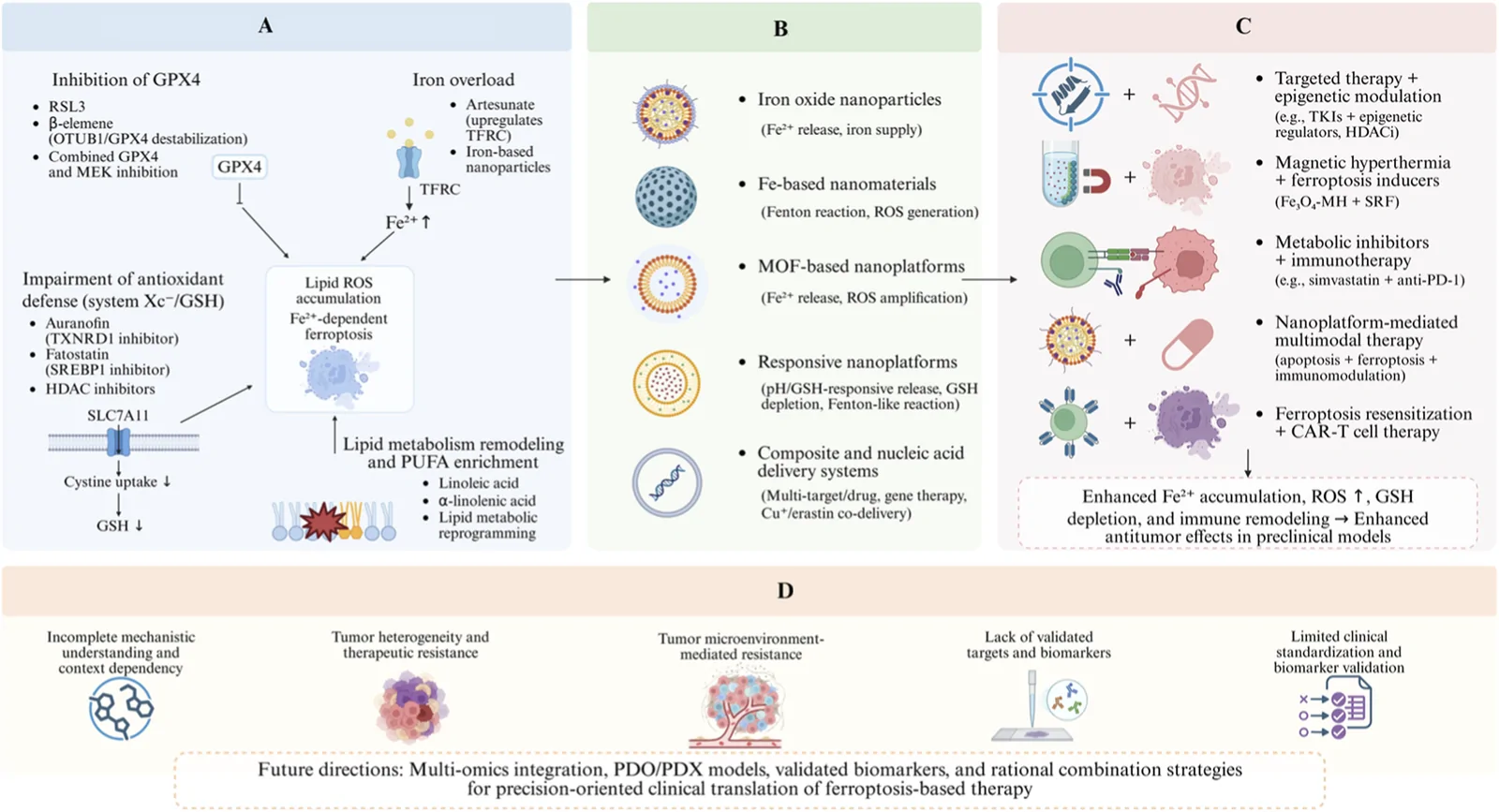
Ferroptosis-targeted therapeutic strategies and translational challenges in digestive system malignancies. GPX4 inhibition, System Xc^−^–GSH antioxidant axis disruption or intracellular iron retention all promote lipid ROS burden and induce an iron-dependent cell death synergistically via ferroptosis. They include iron-based nanomaterials, MOFs, stimulus-responsive nano-platforms and composite delivery systems that can lead to selective accumulation in tumor tissues, controlled release of iron ions for mediated signal amplification of Fenton or Fenton-like reactions, depletion of GSH levels or other combination delivery approaches such as co-delivery of drugs or nucleic acids. Combination approaches will associate ferroptosis induction with various other therapeutic modalities such as targeted or epigenetic therapy, MH, metabolic interventions (in which antioxidants are included), immunotherapy multimodal nanoplatforms and CAR-T-cell therapies to enhance oxidative insult towards modulation of the antitumor immune environment and potentially overcome resistance to therapy. Impediments to translating these promising agents are incomplete mechanistic understanding, context dependent effects, tumor heterogeneity and microenvironment-mediated resistance mechanisms, poorly defined targets and biomarkers of response, and limited standardization in clinical development. Future research directions should focus on multi-omics-based characterization, patient-derived models, candidate predictive biomarkers, and rational combination regimens to enable patient stratification and the translational evaluation of ferroptosis-targeted strategies. Abbreviations: CAR-T, chimeric antigen receptor T-cell; GPX4, glutathione peroxidase 4; GSH, glutathione; MOFs, metal-organic frameworks; PUFA, polyunsaturated fatty acid; ROS, reactive oxygen species; System Xc^−^: cystine/glutamate antiporter system. **(A)** Mechanisms of ferroptosis induction. **(B)** Nanomaterials and delivery systems. **(C)** Combination therapeutic strategies. **(D)** Challenges in clinical translation.

Mechanistically, ferroptosis-inducing agents investigated to date, predominantly in preclinical settings, act through several interconnected mechanisms, including GPX4 inhibition, impairment of antioxidant defenses, intracellular iron accumulation, and lipid metabolic remodeling. It is important to position GPX4 as the key enzyme that detoxifies phospholipid hydroperoxides. Inhibition of the activity or stability of GPX4 results in insufficient reduction of membrane lipid peroxides, and actively promotes ferroptotic cell death (Zhang et al., 2024). While the canonical GPX4 inhibitor RSL3 has primarily been used as a tool compound in laboratory experiments, it is also known to enhance ferroptosis sensitivity of sorafenib-resistant HCC cells in preclinical studies. These observations provide a mechanistic rationale to consider GPX4 as a therapeutic vulnerability; however, clinically useful GPX4 inhibitors with established safety profiles and required potency have not yet been identified (Tang et al., 2025). In addition to that, recent experimental studies have demonstrated that co-inhibiting GPX4 and MEK sequentially leads towards greater production of ROS and increased ferroptotic responses in specific types of malignancies. Although these provide a rationale for the exploration of combinations, their therapeutic potential and safety need to be investigated in more clinically relevant models and defined by prospective trials (Jiang H. et al., 2025). In addition to direct inhibition of enzyme activity, modulation of GPX4 stability is also an interesting mechanistic approach that will merit further pharmacological feasibility and clinical validation. For example, β-elemene disrupts the OTUB1–GPX4 interaction, promotes GPX4 ubiquitination and degradation, and consequently enhances lipid peroxidation and ferroptosis (He et al., 2024). Despite strong mechanistic evidence, direct pharmacological GPX4 inhibition remains primarily a preclinical strategy, and clinically suitable GPX4 inhibitors with acceptable pharmacokinetic and safety profiles have yet to be established.

Metabolic reprogramming of iron metabolic status is another key mechanism of ferroptosis induction. Artesunate functions to increase intracellular iron and labile iron pool content by stabilizing TFRC protein from lysosomal degradation, thereby increasing lipid peroxidation and inducing ferroptosis that attenuates GC cell viability and inhibits tumor growth (Liu Y. et al., 2025). Therefore, targeting iron uptake and homeostasis may enhance ferroptotic vulnerability by increasing intracellular iron-dependent oxidative stress and promoting the transition from tumor-cell survival toward ferroptotic death.

Similarly, attenuation of antioxidant defense systems is key to promoting ferroptosis. Lipid redox homeostasis is maintained by the key barrier System Xc^−^–GSH–GPX4 axis. In particular, SLC7A11 is responsible for cystine uptake and promotion of GSH synthesis; the disruption of this axis dramatically decreases the detoxification of lipid peroxides at the cellular level. The TXNRD1 inhibitor auranofin induces ferroptosis by inhibiting SLC7A11-dependent antioxidant pathways (Lu et al., 2025), whereas the SREBP1 inhibitor fatostatin promotes ferroptosis by decreasing GPX4 expression and increasing lipid peroxidation (Cao et al., 2024a). Moreover, HDAC inhibitors epigenetically modulate lipid metabolism genes and promote lipid peroxidation to enhance the susceptibility of tumors to ferroptosis. Ferroptotic sensitivity is also affected by lipid availability. PUFAs have been reported to increase oxidizable lipid substrates and support ferroptosis-mediated death in pancreatic cancer cells such as those treated with linoleic acid and α-linolenic acid (Suda et al., 2024).

All the ferroptosis inducers discussed above exhibit antitumor activities mainly through perturbation of iron metabolism, lipid metabolism and redox homeostasis. Such agents may directly block GPX4, promote intracellular iron overload, impair SLC7A11-mediated antioxidant capacity, augment oxidizable lipid substrates or manipulate lipid metabolic pathways to induce ferroptosis. Future studies should better define tumor molecular features and resistance states associated with ferroptosis susceptibility and systematically evaluate combinations of ferroptosis inducers with chemotherapy, targeted therapy, radiotherapy, and immunotherapy to establish their preclinical efficacy, safety, and potential translational relevance.

### 6.2 Nanomaterials and delivery systems

Nanomaterials and delivery systems provide an effective technological foundation for ferroptosis-based cancer therapy. Compared with free drugs, nanoplatforms generally exhibit high drug-loading capacity, structural tunability and enhanced tumor-site accumulation, thereby improving therapeutic efficacy in experimental models (Wang et al., 2021). These properties may enhance drug stability, bioavailability and tumor-selective exposure through microenvironment-responsive release; however, whether such approaches can consistently reduce off-target toxicity in humans remains unknown. Accordingly, nanodelivery systems may function not only as carriers of bioactive molecules but also as active modulators of iron homeostasis, redox balance, and lipid peroxidation, thereby enhancing ferroptosis-associated antitumor effects.

Iron-based nanomaterials represent one of the most representative ferroptosis-oriented nanotherapeutic strategies. Magnetic delivery of iron oxide nanoparticles has been reported to enhance local tumor accumulation and therapeutic effects in selected preclinical models. However, clinical biodistribution, systemic toxicity, immune-cell effects and long-term safety require further investigation (Shubhra, 2023). Mesoporous superparamagnetic iron oxide nanoparticles also act as delivery systems and iron donors, releasing free iron ions to catalyze Fenton reactions, enhance ROS generation and invoke ferroptosis (Yang J. et al., 2024). In addition, iron-based metal–organic frameworks (MOFs), such as MIL-100, liberate Fe^2+^ from structure for ferroptotic cell death (Yang F. et al., 2024). These systems combine drug delivery, iron release, and oxidative stress amplification, thereby sustaining lipid-peroxidation-associated damage.

MOF-based and other multifunctional responsive nanoplatforms can further enhance ferroptosis-oriented therapy through tunable pore structures, high loading capacities, and microenvironment-responsive release profiles (Yan et al., 2025). pH/GSH double-responsive nanoplatforms utilize the acidity of TME and high GSH levels in cancer cells for fast release of active agents, as well as carbon monoxide-mediated ROS production for ferroptosis and apoptosis induction (Yang C. et al., 2024). Also, Cu^+^ released through the third stage of double-layered hollow mesoporous cuprous oxide nanoparticles therapy can stimulate Fenton-like reactions and consume GSH to aggravate oxidative stress and promote ferroptosis (Li Z. et al., 2024). Thus they reinforce pro-oxidant reactions and at the same time attenuate antioxidant buffering capacity, making tumor cells more susceptible to ferroptotic damage.

Composite nanodelivery systems can further improve therapeutic efficacy through multi-target, multi-drug or multi-death-mode synergy. Poly(lactic-co-glycolic acid) (PLGA)-based nanoplatforms enable co-delivery of targeted agents and metallic nanoparticles, thereby amplifying ferroptotic and apoptotic effects through GPX4 inhibition and iron-mediated oxidative reactions (Xuan et al., 2025). Nucleic acid delivery systems also offer new opportunities to overcome ferroptosis resistance. Co-delivery of p53 mRNA and NRF2 siRNA suppresses SLC7A11 expression and depletes GSH, thereby restoring ferroptosis sensitivity in tumor cells (Liao et al., 2026). Furthermore, core–shell nanoparticles capable of co-delivering copper ions and the ferroptosis inducer erastin can simultaneously induce cuproptosis and ferroptosis, producing markedly enhanced antitumor activity and providing a novel framework for combinatorial cell death-based therapy (Li et al., 2024f).

Generally, nanomaterials and delivery systems provide multiplexed strategies for ferroptosis-based therapy of digestive system cancers via enabling targeted delivery, tumor-responsive release, Fenton-like reactions, antioxidant depletion and regulation of main ferroptosis-related regulators including GPX4 and SLC7A11. Nevertheless, the clinical translation of most ferroptosis nanoplatforms is still hampered due to potential improvements in pharmacokinetics, tumor targeting, biosafety, stability, clearance or manufacture consistency.

### 6.3 Combination therapeutic strategies

Combination approaches have become a major focus of preclinical investigation for enhancing ferroptosis-associated antitumor effects and addressing potential limitations of single-agent strategies. Ferroptosis is a multilayered process involving dysregulated iron metabolism, excessive lipid peroxide accumulation, and impaired antioxidant defense, making effective tumor elimination through a single target challenging. Tumor heterogeneity, metabolic adaptation, and an immunosuppressive microenvironment further contribute to therapeutic resistance in digestive system malignancies. Thus, the combination of ferroptosis induction and targeted therapy, metabolic intervention, nanotherapy as well as magnetic hyperthermia (MH), chemotherapy or immunotherapy may synergistically enhance the antitumor effects.

Combining targeted therapy with epigenetic modulation represents one strategy for enhancing ferroptotic responses. Co-administration of tyrosine kinase inhibitors and epigenetic regulators induces ferroptosis via upregulation of HMOX1 expression and increased intracellular Fe^2+^ accumulation. The data support that such combinations may deserve further exploration as they have demonstrated synergistic activity against HCC models and pharmacological monotherapy tends to have limitations (Zheng C. et al., 2023). Moreover, the intracellularly mediated MH together with sorafenib has exhibited better tumor growth inhibition as well as increased ferroptotic responses in preclinical models, and little toxicity was observed in relevant experimental systems. However, although these effects are systemic, immune-cell-mediated and not yet established clinically as therapeutic windows (Tang et al., 2024). These findings suggest that combining targeted agents with local energy-based treatments may enhance ferroptotic responses in selected preclinical contexts.

Combination strategies involving metabolic intervention and immunotherapy have also shown considerable promise. The mevalonate pathway is closely linked to CoQ10 biosynthesis and selenoprotein translation, whereas CoQ10 and selenoproteins such as GPX4 are critical for antioxidant defense. Inhibition of the mevalonate pathway reduces CoQ10 synthesis and suppresses selenoprotein translation, thereby weakening antioxidant capacity and inducing ferroptosis (Cheng Y. et al., 2025). On this basis, simvastatin exerts antitumor activity by inducing ferroptosis and enhancing the cytotoxic function of activated CD8^+^ T cells (Sun et al., 2025). Further studies demonstrate that simvastatin can additionally remodel the tumor immune microenvironment, promote CD8^+^ T-cell recruitment and strengthen antitumor immunity. In preclinical models, the combination of simvastatin with immune checkpoint blockade, including anti-PD-1 antibodies, has been reported to enhance antitumor responses and show synergistic potential in microsatellite-stable GC (Ning et al., 2024). Thus, metabolic inhibitors may not only directly induce ferroptosis but also sensitize tumors to immunotherapy through immune microenvironment remodeling.

Nanoplatform-mediated multimodal therapy expands the therapeutic landscape of ferroptosis induction. Bimetallic MOF nanozymes mimic enzymes in catalytic reactions and promote ROS generation and GSH depletion to initiate ferroptosis. Such systems could also be combined with chemotherapeutic agents and immune checkpoint inhibitors for simultaneous induction of apoptosis and ferroptosis along with tumor immune microenvironment modulation, leading to improved overall anti-tumor efficacy (Liu et al., 2026). Furthermore, ferroptosis resensitization approaches may enhance the efficacy of chimeric antigen receptor T-cell (CAR-T) therapy, offering new possibilities for optimizing cellular immunotherapy in solid tumors (Cheng et al., 2024). A mitochondrion-targeted second near-infrared (NIR-II) modulator mediates immunogenic cell death and supports NIR-II-responsive ferroptosis/immune checkpoint co-regulation with synergistic efficacy; however, its explicit link to ferroptosis is yet incompletely characterized which may serve as a future adjunctive strategy for combinatorial immuno-oncology (Wang M. et al., 2025).

Through increasing intracellular Fe^2+^ burden, enhancing ROS accumulation, depleting GSH, suppressing antioxidant systems such as CoQ10/GPX4 and remodeling the tumor immune microenvironment, combination strategies may serve as an adjuvant approach to ferroptosis-based therapy in digestive system tumors. Future studies should stratify mechanistically promising experimental combinations from combinations that are closer to translational. Specifically, this will require integration of tumor molecular subtype, metabolic profile, immune state and candidate ferroptosis biomarkers to identify patients most likely to respond to specific combinations of ferroptosis inducers with targeted agents, metabolic interventions, nanoplatforms chemotherapy or immunotherapy.

Very importantly, the therapeutic strategies discussed above should not be construed as being on equivalent levels of translational development. The majority of direct ferroptosis-targeted approaches, including experimental GPX4 inhibition remain mostly preclinical or exploratory strategies supported by cell-based and xenograft studies (Hangauer et al., 2017). In contrast, metabolic and immunotherapy-based combinations may be much closer to translation if they include agents or therapeutic platforms already clinically used or in early clinical development; however, the specific contribution of ferroptosis to their therapeutic efficacy and ideal patients are currently unvalidated (Zheng Y. et al., 2023). Future translational studies in these approaches will have to systematically tackle pharmacokinetics and biodistribution, treatment-related and off-target toxicity, tumor selectivity, dose optimization, and biomarker guided patient stratification. Reproducible manufacturing, *in vivo* stability and clearance with accompanying long-term biosafety monitoring will also need careful assessment particularly for nanoplatform-based approaches. Thus, current evidence suggests that ferroptosis-targeted therapy should not be considered a uniform clinically mature therapeutic class, but rather the mechanistically promising experimental treatments and selected strategies with greater translational immediacy viewed along a spectrum of clinical completeness.

### 6.4 Current limitations and challenges in clinical translation

Approaches is still in an early stage of exploration. While mechanistic advances in ferroptosis have been substantial, there are practical hurdles that currently impede the straightforward translation of ferroptosis-related discoveries into clinically translatable and safe treatment modalities. These include the fact that much of the evidence available is predominantly preclinical, the context-dependent variability in ferroptosis sensitivity, complex microenvironment-mediated effects on ferroptosis and antitumor immunity (Kim et al., 2022), insufficient validation of therapeutic targets and predictive biomarkers as well as a lack of standardized approaches for patient selection and treatment-response assessment. Supplementary Table 1 summarizes representative therapeutic strategies with their current translational challenges.

Notably, whilst many ferroptosis-targeted strategies mentioned in this Review are validated mostly through *in vitro* assays, animal models, patient-derived organoid (PDO) models or PDX models, but there is currently limited direct clinical validation. Thus, their clinical relevance remains unestablished and should currently be considered hypothesis-generating concepts. This evidence gap represents a major translational hurdle, as neither efficacy, safety, pharmacodynamic activity, nor reproducibility in patients has been fully established to date. Thus far, prospective clinical trials designed to validate ferroptosis induction as a therapeutic mechanism in human digestive system cancers are limited and ferroptosis-specific clinical endpoints, pharmacodynamic biomarkers and treatment-response and failure criteria remain unstandardized.

A second translational barrier is the context dependence of ferroptosis. The detailed regulatory networks and the functional implications of ferroptosis in tumorigenesis and response to therapy are still underexplored in GC (Wei et al., 2025). In a broader sense, susceptibility to ferroptosis may depend on tumor type, disease stage, metabolic state and microenvironmental conditions. While inducing ferroptosis results in tumor growth inhibition in various experimental models, biological or therapeutic context-dependent adaptive antioxidant responses and metabolic plasticity can impact ferroptotic vulnerability, which limits the reproducibility of single-target approaches. Furthermore, dynamic ferroptosis states are difficult to characterize clinically because their characterization would require matched and longitudinal patient samples. This is particularly pertinent in advanced GC, where collection of paired treatment-naïve primary and metastatic specimens remains difficult; it thus precludes a detailed characterization of either the evolutionary dynamics underpinning therapeutic resistance and/or ferroptosis-associated changes (Chemg et al., 2024).

Resistance to therapy and intratumoral heterogeneity also pose obstacles to the implementation of solutions targeting ferroptosis in the clinic. Ferroptosis evasion has been related to chemoresistance in GC, as evidenced by ferroptosis-related regulatory mechanisms that promote resistance to conventional therapy (Huang C. et al., 2025). Ferroptosis-related molecular signatures resistant to treatment are consistently altered in therapy-resistant GC, which seems linked with oxidative stress and the immune microenvironment of tumors (Yu et al., 2024). But those candidate targets from these studies mostly reside at the screening or functional-validation stage and have not yet undergone enough clinical evidence assessment for therapeutic application. Moreover, the emergence of resistant subclones and adaptive signaling under therapeutic high-pressure may restrict both the durability and wider applicability of single-target ferroptosis-based interventions across disease patients and stages. This will ultimately rely on an improved understanding of the underlying biological context that determines whether a specific cell type is susceptible to ferroptosis, instead of uniformly targeting a single ferroptotic pathway.

Emerging Microenvironment-mediated ferroptosis resistance is yet another significant barrier to translation. *In vivo*, ferroptosis susceptibility can be broadly influenced by the surrounding microenvironment, including stromal cells, immune cells, soluble mediators, as well as hypoxia, redox conditions and exosome-mediated communication. Galectin-1, in GC with peritoneal metastasis, induces ferroptosis resistance by activating PI3K/AKT/NRF2/HO-1 antioxidant pathway (Din et al., 2025). Such microenvironmental effects may in part underlie why ferroptosis inducers with robust activity *in vitro* fail to demonstrate similar therapeutic efficacy against complex *in vivo* tumor ecosystems.

A further significant obstacle is the lack of clinically validated therapeutic targets or predictive biomarkers. While multiple ferroptosis-related molecules such as USP7 and MTTP have shown potential biological or prognostic relevance in preclinical studies (Wang W. et al., 2025; Guan et al., 2024), the relevant detection standards, predictive performance and clinical utility for their implementation in practice are still not well-established at this point. Thus, many of the currently proposed ferroptosis-related biomarkers need to be treated as candidate markers rather than verified tools. Accordingly, a hypothesis-generating framework integrating candidate biomarkers with ferroptosis vulnerability and resistance states is presented in Figure 5.

In short, the primary hurdles for clinical translation of ferroptosis-targeted strategies against malignancies in the digestive system can generally be classified into four aspects: incomplete evidence at a clinical level; large biological and intratumoral heterogeneity; microenvironment- and adaptation-mediated ferroptosis resistance; absence of validated therapeutic targets, predictive biomarkers and standardized-based assessment with no prospect. These limitations currently restrict the direct translation of preclinical ferroptosis-related results into reproducible patient-selection criteria or clinically actionable therapeutic approaches. As such, attempts currently to target ferroptosis should be viewed as experimental strategies requiring thorough assessment of efficacy, safety, pharmacodynamic response and reproducibility in the clinic prior to routine implementation.

## 7 Discussion

Collectively, the insights discussed here lend support to a unifying three-layer model whereby ferroptosis in digestive system tumors is dictated by a common iron–lipid–redox vulnerability axis, and tumor-type-specific resistance programs; and most predictably, biomarker-guided therapeutic pairing. Mechanistically, ferroptosis is a biological state characterized by threshold control and determined by the balance between available intracellular iron stores, lipid peroxidation capacity, antioxidant buffering ability and local environmental cues. Throughout the process, many cancers of the digestive system hijack adaptive alterations of redox homeostasis, iron and lipid metabolism as well as antioxidant defense systems, which together determine ferroptotic sensitivity (Badgley et al., 2020). Therefore, the translational significance of ferroptosis is unlikely to depend on a single universal target, but rather on identifying context-specific vulnerabilities and dominant resistance mechanisms. Importantly, ferroptosis-related findings should be interpreted according to evidentiary strength, distinguishing causal regulators from supportive markers and associative observations.

Concerning the first question raised in Introduction, CRC, HCC, GC and PDAC as aforementioned share an evolutionarily conserved iron-lipid-redox ferroptotic machinery. However, the regulatory programs governing this machinery differ substantially among these cancers, thereby determining their context-dependent sensitivity or resistance to ferroptosis. Metabolically plastic and chemotherapy-resistant mechanisms drive CRC, NRF2/GPX4 -related antioxidant adaptation alongside sorafenib resistance in HCC, SLC7A11–GSH–GPX4 signaling pathway activation as well as PI3K/AKT–NRF2 signaling cooperation in GC respectively KRAS-associated metabolic vulnerability with compensatory antioxidant protection in PDAC. Therefore, ferroptosis is a systemic biological weakness that evolves with cancer-specific escape pathways.

In regard to the last question, the divergent or ostensibly opposite effects of ferroptosis are likely substantially dependent on tumor type, cellular compartment, treatment context, experimental model and TME burden. Induction of ferroptosis in tumor cells can limit tumor growth and promote therapeutic sensitivity, but hypoxic conditions, stromal signaling that involves exosome-mediated communication between cells, immune regulation, as well as adaptive antioxidant responses may change or override these effects. Ferroptotic tumor cells may also remodel the surrounding microenvironment. Consequently, ferroptosis needs to be viewed not simply as a universally beneficial therapeutic phenomenon; rather, treatment regimens should be tailored to the prevailing biological setting and resistance mechanism.

As for the third question, many ferroptosis-related biomarkers and therapies are mainly supported by mechanistic and pre-clinical evidence, while prospective validation in clinical cohorts is still rare. Thus, biomarker development should explicitly incorporate the evidence on iron and lipid metabolism, antioxidant capacity, tumor genotype, immune contexture, and TME-associated signals into one larger framework. Integrating multi-omics profiles and single-cell and spatial analyses with validation in PDO/PDX models and independent clinical cohorts will be key to uncovering clinically relevant ferroptosis phenotypes (Cheng et al., 2024).

Thus, Figure 5 should be regarded as a state model generating hypotheses rather than a validated molecular classification. The proposed ferroptosis-sensitive, antioxidant-defense-high, and TME-associated ferroptosis-resistant states are constructs of evidence synthesis, not data-derived molecular clusters or clinically validated subtypes. They are not regarded as mutually exclusive and no standardized quantitative thresholds have currently been established. Future studies should prespecify biomarker panels and thresholds, and ascertain whether these putative states predict pharmacodynamic ferroptosis, principles of treatment response/resistance evolution, and clinical outcome.

**FIGURE 5:**
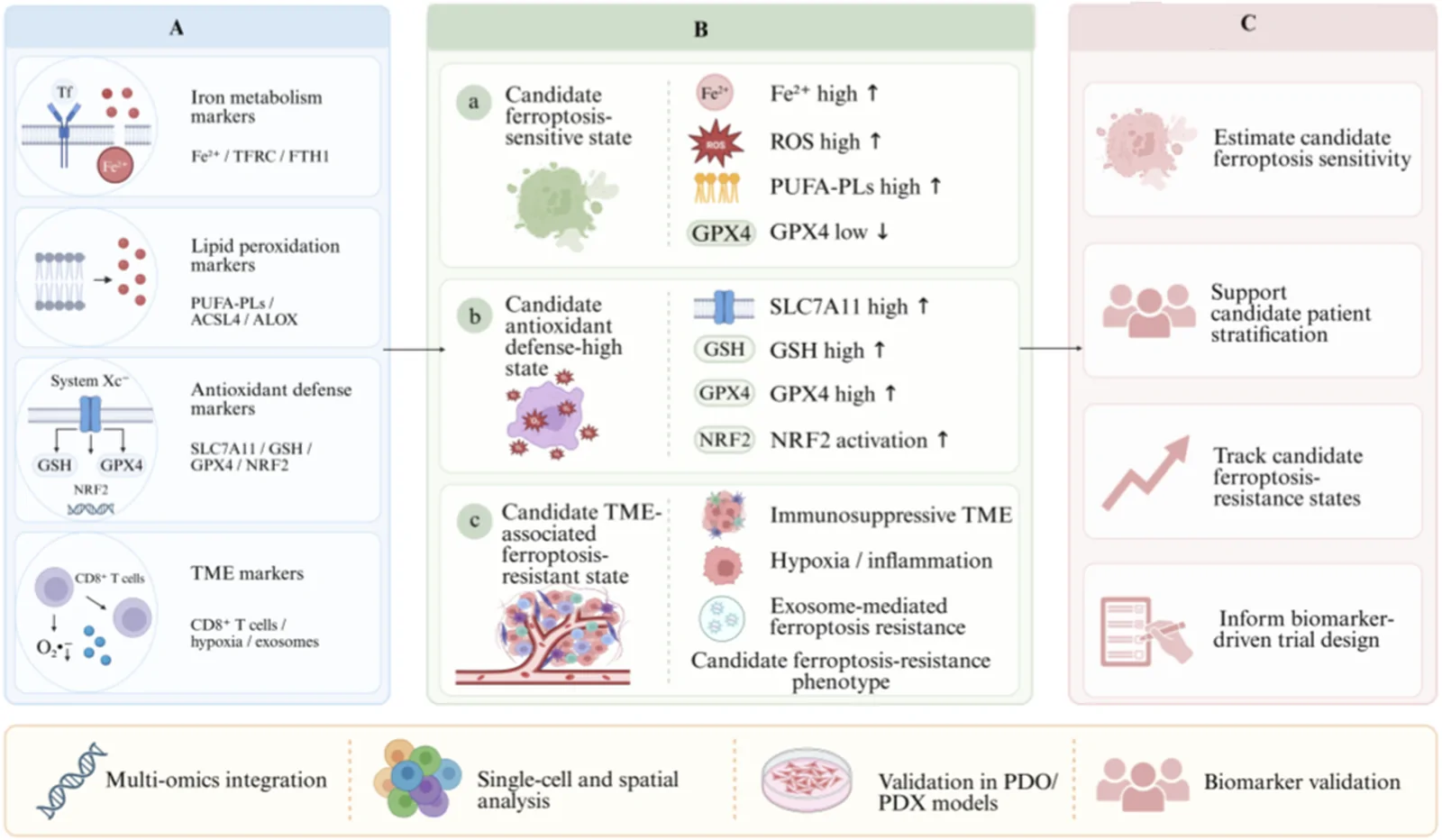
Hypothesis-generating framework of ferroptosis vulnerability and resistance states for future biomarker-guided evaluation. A mechanistic and translational model of conceptual states is examined in this review, though it has not been subjected to clinical validation. Candidate biomarkers are divided into four interrelated biological domains: iron metabolism, lipid-peroxidation potential, antioxidant defense and TME. Biomarker signatures can help with the exploratory assignment of three biological states: a ferroptosis-sensitive state, a high-antioxidant defense state and a TME-associated ferroptosis-resistant state. Some of these states are not distinct molecular subtypes but rather conceptual, and may be spatially contiguous or arise along tumor evolution trajectories or in response to treatment duration. Increased labile Fe^2+^, ROS, and oxidizable PUFA‐PLs, together with reduced GPX4‐mediated antioxidant protection, are directionally consistent with a ferroptosis-sensitive state. In contrast, sustained activation of the SLC7A11–GSH–GPX4 axis, NRF2 signaling, and other redox‐protective programs are consistent with a candidate antioxidant‐defense-high state, whereas hypoxia, inflammation, immunosuppression, stromal signaling, and exosome-mediated communication characterize a candidate TME‐associated ferroptosis‐resistant state. For the moment, tumors cannot be classified into these categories as there are no validated quantitative thresholds. Thus, this framework serves as a set of biomarkers to generate testable or potentially falsifiable hypotheses for biomarker development going forth—rather than a definitive system able to define patient stratification. Prospective validation requires pre-specification of biomarker panels and cut-offs, establishing the association of candidate states in independent patient cohorts and patient-derived models with pharmacodynamic evidence of ferroptosis, therapeutic response, lineage evolution, and clinical outcome. Abbreviations: Fe^2+^, ferrous ion; ROS, reactive oxygen species; PUFA-PLs, polyunsaturated fatty acid-containing phospholipids; GPX4, glutathione peroxidase 4; SLC7A11, solute carrier family 7 member 11; GSH, glutathione; NRF2, nuclear factor erythroid 2-related factor 2; TME, tumor microenvironment. **(A)** Biomarker inputs. **(B)** Hypothesis-generating ferroptosis sensitivity framework. **(C)** Potential translational applications.

## Glossary

AA: arachidonic acid
ACE: acevaltrate
ACSL4: acyl-CoA synthetase long-chain family member 4
AdA: adrenic acid
AKR1C1: aldo-keto reductase family 1 member C1
ALOX: arachidonate lipoxygenase
ASO: antisense oligonucleotide
ATF2: activating transcription factor 2
BSO: buthionine sulfoximine
CAF: cancer-associated fibroblast
CAR-T: chimeric antigen receptor T-cell
CoQ10: coenzyme Q10
CRC: colorectal cancer
CREB: cAMP response element-binding protein
ELOVL5: elongation of very long-chain fatty acids protein 5
ESCC: esophageal squamous cell carcinoma
FADS2: fatty acid desaturase 2
FNR: ferroptosis negative regulatory axis
FSP1: ferroptosis suppressor protein 1
FTH1: ferritin heavy chain 1
GAC: gastric adenocarcinoma
GATA3: GATA-binding protein 3
GC: gastric cancer
GCLC: glutamate–cysteine ligase catalytic subunit
GEM: gemcitabine
GPX4: glutathione peroxidase 4
GSH: glutathione
HCC: hepatocellular carcinoma
HDAC: histone deacetylase
HMOX1: heme oxygenase 1
HO-1: heme oxygenase-1
IKE: Imidazole ketone erastin
KDM6B: lysine demethylase 6B
KEAP1: kelch-like ECH-associated protein 1
KRAS: kirsten rat sarcoma viral oncogene homolog
LCN2: lipocalin 2
LDHA: lactate dehydrogenase A
LPCAT2/3: lysophosphatidylcholine acyltransferase 2/3
m6A: N6-methyladenosine
MDA: malondialdehyde
MDM2: mouse double minute 2 homolog
METTL3: methyltransferase-like 3
MH: magnetic hyperthermia
MOFs: metal–organic frameworks
MTTP: microsomal triglyceride transfer protein
MYDGF: myeloid-derived growth factor
NIR-II: second near-infrared
NRF2: nuclear factor erythroid 2-related factor 2
ODC1: ornithine decarboxylase 1
OGT: O-GlcNAc transferase
OTUB1: OTU deubiquitinase, ubiquitin aldehyde binding 1
PCBP: poly(rC)-binding protein
PDAC: pancreatic ductal adenocarcinoma
PDO: patient-derived organoid
PDX: patient-derived xenograft
PRAP1: proline rich acidic protein 1
PRDX1: peroxiredoxin 1
PRMT1: protein arginine methyltransferase 1
PUFA-PLs: PUFA-containing phospholipids
PUFAs: polyunsaturated fatty acids
ROS: reactive oxygen species
RSL3: ras-selective lethal 3
SAS: sulfasalazine
SCD: stearoyl-CoA desaturase
SLC3A2: solute carrier family 3 member 2
SLC7A11: solute carrier family 7 member 11
**System Xc** ^**-**^: cystine/glutamate antiporter system
TAX1BP1: Tax1-binding protein 1
TCF4: transcription factor 4
TFRC: transferrin receptor 1
TME: tumor microenvironment
TMOD3: tropomodulin 3
USP7/8: ubiquitin-specific protease 7/8
VKORC1L1: vitamin K epoxide reductase complex subunit 1-like 1
XBP1s: spliced X-box binding protein 1
YBX1: Y-box-binding protein 1
ZEB1: zinc finger E-box-binding homeobox one
ZIP8: Zrt- and Irt-like Protein 8
