# Supplementary material for: Targeting ferroptosis in major digestive system malignancies: regulatory networks and therapeutic opportunities
Source: Front Cell Dev Biol. 2026 Oct 29;14:1945918. doi: 10.3389/fcell.2026.1945918 (PMC13755419; doi:10.3389/fcell.2026.1945918)
Supplement: Supplementary file 1 [file Table1.docx]

**Supplementary table 1. Ferroptosis-Targeted Therapeutic Strategies in Digestive System Tumors: Regulatory Mechanisms and Translational Challenges**

| **Category of Therapeutic Strategies** | **Representative Interventions/Agents** | | **Key Regulatory Targets/Pathways** | | **Mechanisms of Ferroptosis Regulation** | **Major Biological Effects** | **Relevance to Antitumor Therapy** | **Evidence level/model system** | **Clinical Translation Status and Challenges** | **References** |
| --- | --- | --- | --- | --- | --- | --- | --- | --- | --- | --- |
| Deubiquitination-mediated ferroptosis-targeting strategy | | USP8 inhibitors (e.g., DUB-IN-2) combined with ferroptosis inducers (e.g., erastin, SAS, or IKE) | | USP8–GPX4 axis; System Xc⁻/GSH/GPX4 pathway | USP8 stabilizes GPX4 via deubiquitination; its inhibition promotes proteasomal degradation of GPX4, leading to increased lipid peroxidation and ferroptosis sensitivity | Increased lipid peroxidation; enhanced ferroptosis sensitivity; increased CD8⁺ T-cell infiltration | Suppresses tumor growth; enhances anti-PD-1 immunotherapy efficacy; synergizes with ferroptosis inducers | Preclinical mechanistic evidence; CRC cell models, AOM/DSS-induced autochthonous CRC in IEC-specific Usp8-deficient mice, and CT26 syngeneic tumors with ferroptosis/anti-PD-1 combination validation. | Potential toxicity due to the role of USP8 in normal tissue homeostasis; requires optimization of dosage and safety | ^[62]^ |
| Metal ion-mediated ferroptosis-targeting strategy | | Cu²⁺; copper supplementation (copper gluconate); copper chelators (TM; TEPA); combination with erastin/IKE | | GPX4; Tax1-binding protein 1 (TAX1BP1)-mediated autophagy pathway | Copper directly binds to GPX4, promoting its aggregation and ubiquitination, leading to TAX1BP1-dependent autophagic degradation of GPX4 and subsequent lipid peroxidation and ferroptosis | Increased lipid peroxidation; enhanced ferroptosis sensitivity | Copper enhances the antitumor efficacy of erastin/IKE and promotes tumor suppression in pancreatic cancer models | Preclinical mechanistic evidence; human PDAC cell models and PANC-1 subcutaneous xenografts in nude mice with copper/IKE treatment validation. | Copper exhibits dual roles (pro-ferroptotic versus protective via chelation); safety and context-dependent effects remain challenges | ^[144]^ |
| Small molecule–mediated ferroptosis-targeting strategy (dual-target mechanism) | | ACE | | PCBP1/2 (iron chaperones); GPX4 | ACE increases intracellular Fe²⁺ by downregulating PCBP1/2 and disrupts the antioxidant system by directly binding to GPX4, inhibiting its activity and promoting its ubiquitin-mediated degradation, leading to lipid peroxidation and ferroptosis | Increased Fe²⁺ accumulation; enhanced lipid peroxidation; induction of ferroptosis; inhibition of cell proliferation and migration | Suppresses colorectal tumor growth in vitro and in vivo; shows superior efficacy compared to conventional ferroptosis inducers and first-line drugs; effective in tumor organoids | Preclinical mechanistic evidence; RKO/HCT116 CRC cells, oxaliplatin-resistant HCT116 cells, RKO/HCT116-luc xenografts, human CRC organoid models, and human tissue validation. | Potential safety concerns of ferroptosis induction (e.g., organ injury) require further validation, although no significant toxicity observed in current models | ^[22]^ |
| Ferroptosis resistance-targeting strategy (GPX4-dependent) | | GPX4 depletion or pharmacologic inhibition of ferroptosis resistance; combination with CAR-T therapy | | GPX4-mediated ferroptosis tumor pathway | Upregulation of GPX4 enables cancer cells to evade ferroptosis during metastatic progression; depletion or inhibition of GPX4 restores ferroptosis sensitivity | Suppression of tumor growth and metastatic progression | Enhances antitumor efficacy; ferroptosis resensitization augments CAR-T therapy response | Translational/preclinical evidence; paired primary–metastatic patient single-cell profiling, GAC cell lines, PDX/CDX models, autochthonous KPCS/KPCS;Gpx4 mice, and patient-derived peritoneal metastasis xenografts with IKE–CAR T validation | Lack of specific clinically validated GPX4-targeting agents; challenges in safely targeting ferroptosis in patients | ^[10]^ |
| Transcriptional regulation–mediated ferroptosis-targeting strategy (STAT3-dependent) | | Genetic inhibition of STAT3; pharmacological STAT3 inhibitor (W1131); combination with 5-FU or erastin | | STAT3; GPX4; SLC7A11 (System Xc⁻); FTH1 | STAT3 transcriptionally upregulates ferroptosis negative regulators (GPX4, SLC7A11, FTH1); inhibition of STAT3 suppresses these genes, leading to Fe²⁺ accumulation, GSH depletion, lipid peroxidation, and induction of ferroptosis | Increased Fe²⁺ levels; lipid ROS accumulation; GSH depletion; enhanced lipid peroxidation | Suppresses gastric tumor growth; alleviates chemoresistance; synergizes with 5-FU and ferroptosis inducers | Preclinical/translational evidence; GC patient specimens, AGS/MGC803 and 5-FU-resistant MGC803/5-FU cells, CDX/PDX models, and PDX-derived organoids with W1131/5-FU treatment validation | No approved STAT3 inhibitors for GC; safety and efficacy of ferroptosis induction require further validation | ^[134]^ |
| Epigenetic regulation–mediated ferroptosis-targeting strategy (histone lactylation-dependent) | | p300 inhibitor; lactate dehydrogenase A (LDHA) inhibitor (oxamate); glutamate–cysteine ligase catalytic subunit (GCLC) inhibitor (buthionine sulfoximine [BSO]); combination with oxaliplatin | | p300; LDHA; HDAC1; H4K12 lactylation; GCLC; GPX4 | p300 catalyzes H4K12 lactylation, which transcriptionally upregulates GCLC, enhancing GSH synthesis and inhibiting ferroptosis; inhibition of p300, LDHA, or GCLC reduces H4K12 lactylation or GCLC expression, leading to increased lipid peroxidation and ferroptosis | Decreased lipid ROS and MDA under lactylation activation; increased lipid ROS and MDA upon inhibition; modulation of GSH synthesis | Enhances chemoresistance in CRC stem cells; targeting this axis sensitizes tumors to oxaliplatin and promotes ferroptosis-mediated tumor suppression | Preclinical mechanistic evidence with human correlative validation; CRC patient tissues/scRNA-seq datasets, LoVo/SW620/HCT116 CSC models including oxaliplatin-resistant CCSCs, and LoVo CSC-derived xenografts with p300/LDHA/GCLC-targeting validation | GCLC inhibitor (BSO) has been evaluated in clinical settings for other tumors; safety and efficacy in CRC require further validation | ^[48]^ |
| Signaling pathway–mediated ferroptosis resistance-targeting strategy (Wnt/β-catenin–transcription factor 4 [TCF4] axis) | | Wnt/β-catenin inhibitor (LF3); TCF4 depletion/knockout; combination with cisplatin | | Wnt/β-catenin signaling; TCF4; GPX4 | Activation of Wnt/β-catenin signaling promotes formation of the β-catenin/TCF4 transcription complex, which directly binds to the GPX4 promoter and upregulates its expression, thereby reducing lipid peroxidation and inhibiting ferroptosis; inhibition of this pathway suppresses GPX4 and restores ferroptosis sensitivity | Decreased lipid ROS, MDA, and 4-HNE under pathway activation; increased lipid peroxidation upon inhibition; enhanced ferroptosis sensitivity | Enhances sensitivity to cisplatin; suppresses tumor growth in vitro and in vivo; overcomes chemotherapy resistance in GC | Preclinical mechanistic evidence with human correlative validation; GC patient tissues, AGS/HGC-27/MKN-45 cells, and MKN-45 xenografts with TCF4-targeting/LF3–cisplatin validation | Wnt pathway inhibitors not yet clinically approved; potential toxicity due to pathway roles in normal tissues | ^[11]^ |
| TME–mediated ferroptosis regulation (adipocyte-derived exosome signaling) | | Adipocyte-derived exosomes carrying microsomal triglyceride transfer protein (MTTP); MTTP knockdown | | MTTP; proline rich acidic protein 1 (PRAP1); zinc finger E-box-binding homeobox 1 (ZEB1); GPX4; xCT (SLC7A11); lipid metabolism pathways | Exosomal MTTP forms a complex with PRAP1 to suppress ZEB1 expression, leading to upregulation of GPX4 and xCT, reduction of PUFA levels, and decreased lipid peroxidation, thereby inhibiting ferroptosis | Decreased lipid ROS and MDA levels; increased GSH; reduced PUFA content; suppression of ferroptotic cell death | Suppresses ferroptosis and promotes oxaliplatin chemoresistance in CRC; targeting MTTP restores ferroptosis sensitivity and enhances chemotherapy efficacy | Translational/preclinical evidence; CRC patient plasma/tissues, HCT116/SW480 adipocyte-exosome models, patient-derived CRC organoids, and ob/ob mouse tumor models with MTTP-targeting/oxaliplatin validation | Validated in organoid models and obese mouse tumor models; challenges include targeting exosome-mediated signaling and metabolic heterogeneity in patients | ^[145]^ |
| Small molecule–induced ferroptosis strategy (natural flavonoid compound) | | Baicalein (5,6,7-trihydroxyflavone) | | JAK2; STAT3; GPX4 | Baicalein directly binds to JAK2 and inhibits JAK2 phosphorylation, thereby suppressing STAT3 activation and transcriptional regulation of GPX4, leading to decreased GPX4 expression, increased lipid peroxidation, and induction of ferroptosis | Increased ROS, MDA, and Fe²⁺ levels; decreased GSH and mitochondrial membrane potential; lipid peroxidation and ferroptotic cell death | Inhibits CRC cell proliferation and tumor growth via ferroptosis induction; anti-tumor effect validated in xenograft models | Preclinical mechanistic evidence; HCT116/DLD1 CRC cells and BALB/c nude-mouse subcutaneous CRC xenografts with baicalein treatment validation | Validated in vitro and in vivo; further studies needed on pharmacokinetics, safety, and clinical efficacy | ^[146]^ |
| Metabolic dependency–targeting ferroptosis sensitization strategy | | Glutamine antagonist (DON); combination with GPX4 inhibitor (RSL3) | | Glutamine metabolism; PAXIP1; H3K4me3; HMOX1; GPX4; SLC7A11 | Glutamine deprivation or DON treatment induces PAXIP1-mediated H3K4me3 upregulation, activating transcription of ferroptosis-related genes (including HMOX1, GPX4, and SLC7A11); HMOX1 promotes Fe²⁺ accumulation and lipid peroxidation, while adaptive upregulation of GPX4 confers vulnerability to GPX4 inhibitor-induced ferroptosis | Increased lipid peroxidation, MDA, and labile Fe²⁺ levels; activation of ferroptosis program | Sensitizes PDAC cells to GPX4 inhibitor-induced ferroptosis; combination treatment suppresses tumor growth in vitro and in PDX models | Preclinical mechanistic/translational evidence; murine and human PDAC cell models, subcutaneous tumors, and two independent PDAC PDX models with DON–RSL3 combination validation | Validated in PDX models; glutamine metabolism inhibitor prodrug (DRP-104) in early clinical trials; lack of clinically approved GPX4 inhibitors and potential toxicity remain challenges | ^[3]^ |
| Deubiquitination-targeted ferroptosis induction strategy | | USP7 inhibitor | | USP7; stearoyl-coa desaturase (SCD) | USP7 inhibition promotes ubiquitination and degradation of SCD, disrupting lipid metabolism and leading to increased lipid peroxidation and ferroptosis induction | Increased lipid ROS, MDA accumulation, iron overload, and mitochondrial damage | Suppresses GC cell proliferation, migration, invasion, tumor growth, and metastasis via ferroptosis induction | Translational/preclinical evidence; human GC tissues, multiple GC cell models, MGC803-luc/MKN1-luc orthotopic xenografts, and DHPO-treated GC PDX models | Validated in vitro and in orthotopic and PDX models; no USP7 inhibitors have entered clinical trials; challenges include drug development and specificity | ^[128]^ |
| TME–mediated ferroptosis suppression strategy (CAF-derived exosomal miRNA) | | CAF-derived exosomes carrying miR-3173-5p | | miR-3173-5p; ACSL4 | Exosomal miR-3173-5p derived from CAFs targets and suppresses ACSL4 expression in pancreatic cancer cells, leading to reduced lipid peroxidation and inhibition of ferroptosis | Decreased lipid ROS and intracellular Fe²⁺ levels; reduced ferroptotic cell death | Suppresses ferroptosis and induces GEM resistance in PDAC | Preclinical/translational evidence; patient-derived CAFs/PDAC tissues, PANC-1/BxPC-3 CAF-exosome models, and PANC-1 xenografts with GEM-response validation | Validated in xenograft models; challenges include targeting exosome-mediated signaling and stromal–tumor interactions | ^[133]^ |
| Ferroptosis evasion–targeting strategy (protein lactylation–NRF2 axis) | | ZNF207 knockdown; disruption of peroxiredoxin 1 (PRDX1) lactylation; inhibition of NRF2 activity | | ZNF207; PRDX1 (lactylation at K67); NRF2 | ZNF207 promotes lactylation of PRDX1, enhancing its nuclear translocation and activation of NRF2, which induces antioxidant responses and suppresses ferroptosis | Enhanced antioxidant response; reduced ferroptosis | Promotes resistance to regorafenib; targeting the ZNF207–PRDX1–NRF2 axis restores ferroptosis and enhances drug sensitivity | Preclinical mechanistic evidence; MHCC-97L/Li-7 HCC cells with CRISPR/Cas9 screening and ZNF207–PRDX1–NRF2 perturbation for RGF-resistance/ferroptosis validation | Lack of clinically available inhibitors targeting this axis and need for further validation | ^[30]^ |
| Ferroptosis resistance-targeting strategy (ubiquitination–NMD–GPX4 axis) | | TRIM34 knockdown | | TRIM34; UPF1; GPX4 | TRIM34 promotes ubiquitination and degradation of UPF1, thereby suppressing GPX4 mRNA degradation and increasing GPX4 protein levels, leading to inhibition of ferroptosis | Suppressed ferroptosis; enhanced proliferation, migration, and invasion | Targeting TRIM34 restores ferroptosis sensitivity and enhances response to anti-PD-1 immunotherapy in HCC | Preclinical mechanistic evidence with human correlative validation; Huh7 CRISPR/Cas9 ferroptosis screen, TRIM34–UPF1–GPX4 HCC cell models, and TRIM34-knockdown tumor models with anti-PD-1 validation | Lack of clinically available inhibitors targeting TRIM34 and need for further validation | ^[147]^ |
| Metabolic dependency–targeting ferroptosis sensitization strategy (glutamine transport axis) | | SLC38A5 knockdown/inhibition | | SLC38A5; glutamine metabolism; NRF2; GPX4; mTOR-SREBP1-SCD1 pathway | Inhibition of SLC38A5 reduces glutamine uptake, leading to decreased GSH levels and downregulation of NRF2 and GPX4, as well as suppression of mTOR-SREBP1-SCD1 signaling, resulting in increased lipid ROS accumulation and induction of ferroptosis | Increased ROS and lipid ROS levels; decreased GSH; mitochondrial dysfunction; enhanced ferroptotic cell death | Restores GEM sensitivity and suppresses tumor growth and metastasis in pancreatic cancer | Preclinical mechanistic evidence with human correlative validation; GEM-sensitive/resistant PDAC tissues, PANC-1/Capan-1 and PANC-GR/Capan-GR cells, and PANC-1 orthotopic xenografts with SLC38A5-targeting/ferroptosis validation | Lack of clinically available SLC38A5-targeting agents and need for further validation | ^[148]^ |
| Epigenetic/transcriptional regulation–mediated ferroptosis induction strategy | | Protein arginine methyltransferase 1 (PRMT1) inhibitor (C-7280948); SLC7A11 inhibitor (HG106); lysophosphatidylcholine acyltransferase 2 (LPCAT2) overexpression | | LPCAT2; PRMT1; SLC7A11 | LPCAT2 induces acetylation of PRMT1 at K145 and retains PRMT1 in the cytoplasm, thereby reducing PRMT1-mediated activation of the SLC7A11 promoter and decreasing SLC7A11 expression, which leads to induction of ferroptosis | Increased ROS and MDA levels; decreased GSH; mitochondrial shrinkage and membrane density changes | Inhibits CRC cell proliferation and tumor progression; PRMT1 or SLC7A11 inhibition delays tumor development in vivo | Preclinical mechanistic evidence with human correlative validation; CRC tissues/scRNA-seq, LPCAT2-manipulated CRC cells, subcutaneous CDX models, and AOM/DSS-induced CRC in LPCAT2−/− mice with PRMT1/SLC7A11 inhibitor validation | Preclinical stage; validated in xenograft and mouse models; no clinical validation reported | ^[149]^ |
| Microenvironment metabolite–mediated ferroptosis sensitization strategy | | Butyrate supplementation; combination with erastin or oxaliplatin | | c-Fos; SLC7A11 (xCT); class I HDAC | Butyrate inhibits class I HDAC activity, leading to induction of c-Fos expression, which suppresses SLC7A11 (xCT) expression and reduces xCT-dependent GSH synthesis, thereby enhancing ferroptosis sensitivity | Increased ROS and lipid peroxidation; decreased GSH; enhanced ferroptotic cell death | Enhances the antitumor efficacy of ferroptosis inducers and oxaliplatin; overcomes ferroptosis resistance, including in cancer stem cells | Translational/preclinical evidence; CRC patient specimens/metabolomics, CRC cell and CSC models, patient-derived CRC organoids, HCT116/SW480 xenografts, and AOM/DSS CRC models with butyrate–erastin/OXA combination validation | Validated in mouse models, organoids, and patient samples | ^[150]^ |
| Metal ion transport–targeted ferroptosis modulation strategy | | nobiletin (ZIP8 inhibitor); ZIP8 knockdown | | ZIP8 (SLC39A8); Zn²⁺; cAMP response element-binding protein (CREB) phosphorylation; GPX4 | ZIP8 enhances intracellular Zn²⁺ levels, promoting CREB phosphorylation, which upregulates GPX4 expression and inhibits ferroptosis; inhibition of ZIP8 reduces GPX4 and restores ferroptosis | Increased lipid peroxidation and Fe²⁺ accumulation; decreased GPX4 expression; enhanced ferroptosis | Targeting ZIP8 or using nobiletin suppresses ESCC cell proliferation and tumor growth by promoting ferroptosis | Translational/preclinical evidence; ESCC patient tissues/TCGA-GEO datasets, ZIP8-manipulated human ESCC cells, and Nobiletin-treated ESCC PDX models in SCID mice | Preclinical stage; validated in vitro and in PDX mouse models; further clinical validation required | ^[98]^ |
| Non-coding RNA–mediated ferroptosis suppression and resistance-targeting strategy | | LncRNA-HMG inhibition using antisense oligonucleotide (ASO) | | LncRNA-HMG; p53; mouse double minute 2 homolog (MDM2); SLC7A11; vitamin K epoxide reductase complex subunit 1-like 1 (VKORC1L1) | LncRNA-HMG binds to p53 and promotes MDM2-mediated ubiquitination and degradation of p53, leading to upregulation of SLC7A11 and VKORC1L1, which enhances reducing capacity, eliminates ROS and lipid peroxides, and suppresses ferroptosis | Decreased ROS and lipid peroxidation; increased GSH/GSSG ratio; reduced ferroptotic cell death | Promotes chemoresistance to 5-FU and oxaliplatin; inhibition of LncRNA-HMG restores ferroptosis and enhances chemotherapy sensitivity | Translational/preclinical evidence; CRC patient cohorts, LoVo/HCT116 and chemotherapy-resistant cell models, LoVo xenografts, and p53-WT CRC PDX models with LncRNA-HMG ASO plus 5-FU/oxaliplatin validation | ASO-based targeting requires further clinical development | ^[67]^ |
